# Your Next State-of-the-Art Could Come from Another Domain: A Cross-Domain Analysis of Hierarchical Text Classification

**DOI:** 10.1007/s10994-025-06993-w

**Published:** 2026-03-31

**Authors:** Nan Li, Bo Kang, Tijl De Bie

**Affiliations:** IDLab, Ghent University, 9052 Ghent, Belgium

**Keywords:** Hierarchical multi-label text classification, Cross-domain analysis, Label hierarchy, Dataset characteristics, Large language models, Survey, Benchmark

## Abstract

Text classification with hierarchical labels is a prevalent and challenging task in natural language processing. Examples include assigning ICD codes to patient records, tagging patents with IPC classes, assigning EUROVOC descriptors to European legal texts, and more. Despite the prevalence of hierarchical text classification problems, a comprehensive understanding of state-of-the-art methods across different application domains has been lacking. In this paper, we propose a unified methodology to break down the boundaries between these different domains, thus enabling cross-domain transfer of innovative ideas. We first construct a *Unified Framework* that translates distinct domain-specific methods into a common architectural language. Applying this framework, we conduct a comprehensive *Cross-Domain Benchmark* that exposes architectural gaps often overlooked in single-domain studies. We then demonstrate the framework’s practical utility through a validation case study, where we synthesize a new state-of-the-art hierarchical text classification method by combining submodules that were developed for the medical and legal domains. Our extensive empirical analysis yields key insights and guidelines, confirming the necessity of cross-domain learning for designing effective methods. Our code and datasets are publicly available at https://github.com/aida-ugent/cross-domain-HTC.

## Introduction

Text classification with hierarchical labels is a fundamental challenge in natural language processing, where the goal is to assign one or more labels from a hierarchically organized label set to each text input. This problem appears across diverse domains and is particularly pronounced in scenarios with large and complex label structures. Examples include medical coding, which assigns International Classification of Diseases (ICD) codes to patient records (Edin et al., 2023); patent classification, which predicts International Patent Classification (IPC) codes for patent documents (Kamateri et al., 2024); and extreme multi-label classification tasks in legal, Wikipedia, and e-commerce domains (Bhatia et al., 2016). While these applications may seem distinct, they share a common core: classifying text with labels that have inherent hierarchical relationships.

Despite this commonality, current research is largely confined within individual domains. Methods are typically developed and evaluated only on domain-specific datasets, with minimal cross-domain analysis or comparison. This domain-centric approach has led to three significant knowledge gaps in the research literature: (1) how methods that excel in one domain compare to domain-specific approaches in others; (2) which architectural components (which we refer to as *submodules*, such as text encoders, label encoders, and prediction mechanisms) are truly domain-specific versus universally effective; and (3) how dataset characteristics, rather than domain origin, influence method performance.

In this work, we use the term “cross-domain” to refer to the application of supervised architectures developed in one discipline (e.g., Legal) to datasets from another (e.g., Medical). This is distinct from unsupervised domain adaptation or transfer learning techniques often associated with the term.

Conducting such an evaluation is critical for the advancement of the field. While methods are often optimized for specific domain characteristics (e.g., long documents in medical texts), evaluating them on diverse datasets is necessary to distinguish truly generalizable architectural innovations from domain-overfitted designs. Such evaluation is inevitable as the field moves towards foundational models, yet it requires a systematic approach to be rigorous.

To address these gaps, we present a unified methodology designed to break down domain isolation. Our approach follows a logical progression: *the Tool* (Framework), *the Diagnosis* (Benchmark), and *the Validation* (Synthesis), yielding the following key contributions:

- **The Tool: Unified Framework & Survey** We propose the first domain-agnostic framework that decomposes hierarchical text classification methods into nine essential submodules. This framework is grounded in a comprehensive **Cross-Domain Survey** of 32 representative methods, translating distinct domain vocabularies into a common architectural language.
- **The Diagnosis: Cross-Domain Benchmark** We conduct the first large-scale cross-domain evaluation to identify architectural gaps. This analysis involves eight state-of-the-art methods across five domains, revealing performance patterns often overlooked in single-domain studies.
- **The Validation: Case Study & New State of the Art** We demonstrate the framework’s practical utility by synthesizing a new model (combining submodules from Medical and Legal domains). This validation yields **New State-of-the-Art Results**^1^ on NYT-166, SciHTC-83, USPTO2M-632, and MIMIC3-3681.
- **Novel Resources: Enhanced Datasets** To enable this rigorous cross-domain evaluation, we provide: (1) a cleaned version of EurLex with recovered taxonomy and restored original text; (2) a new dataset derived from EurLex, EurLex-DC-410; and (3) a carefully curated selection of eight datasets across five domains, with necessary adaptations for fair cross-domain evaluation.
- **Empirical Insights** Our findings through extensive experiments reveal that dataset characteristics and architectural design choices, rather than domain specificity, primarily determine method effectiveness. We encourage researchers to look beyond their specific domains, leveraging innovations from other fields.

The rest of the paper is organized as follows. Section 2 reviews existing surveys and benchmarks, highlighting the need for cross-domain analysis. Section 3 presents our unified framework and formal problem definition, analyzing how different methods utilize label hierarchy information. Section 4 details our cross-domain evaluation, including dataset selection, method descriptions, and experimental results. Section 5 provides an in-depth analysis of our findings, discussing the cross-domain performance of different methods, the impact of several key factors, and lessons for practitioners. Section 6 summarizes key findings and discusses limitations and future directions.

## Related Work

Section 2.1 presents surveys and benchmarks for the general problem of multi-label text classification. Section 2.2 focuses on domain-specific surveys and benchmarks.

### General Surveys and Benchmarks

An overview of multi-label text classification is provided by Chen et al. (2022), which systematically categorizes methods by data efficiency, feature extraction, and label correlation modeling, but focuses on flat classification without exploring hierarchical structures or cross-domain analysis. An empirical comparison across benchmark datasets is presented by Bogatinovski et al. (2022), examining how model performance relates to dataset characteristics, but primarily examines non-textual domains with small label spaces. The work of Wei et al. (2022) focuses on extreme multi-label learning challenges and collects datasets and tools, but does not address hierarchical structures or provide empirical validation. Similarly, Bhatia et al. (2016) maintain a widely cited repository of extreme multi-label classification methods and datasets spanning e-commerce, Wikipedia, and legal domains, though many datasets only contain preprocessed feature vectors without original texts, limiting their applicability for pretrained language models.

Various aspects of multi-label classification are covered in recent surveys: multiple modalities (Han et al., 2023), label imbalance (Tarekegn et al., 2021), and deep learning architectures (Tarekegn et al., 2024), but none comprehensively address text datasets, hierarchical structures, or cross-domain analysis. A review of hierarchical multi-label text classification methods by Liu et al. (2023) categorizes them into tree-based, embedding-based, and graph-based approaches, but covers limited domains and lacks empirical validation.

Most recently, Bertalis et al. (2024) compare hierarchical text classification (HTC) and extreme multi-label classification (XML), evaluating two methods on three datasets from each of these domains. While their observation about XML methods’ adaptability to HTC tasks aligns with one aspect of our findings, our work provides the first comprehensive cross-domain analysis spanning five domains, evaluating eight state-of-the-art methods on eight diverse datasets, with a unified framework for analyzing method components and in-depth empirical analyses of their effectiveness.

### Domain-Specific Benchmarks and Surveys

Complementing the general surveys, several studies provide in-depth analyses and benchmarks within specific application domains, demonstrating the prevalence of HTC tasks but also the tendency for research to remain isolated.

For automatic medical coding, a common HTC task involving assigning ICD codes to patient records, Edin et al. (2023) provide a critical benchmark of state-of-the-art models on cleaned versions of the MIMIC-III and MIMIC-IV datasets. They offer a valuable open-source evaluation pipeline, promoting reproducibility within the medical NLP community. Their work, however, focuses solely on the medical domain, without comparing medical coding methods to approaches from other fields or vice-versa.

In patent classification, Kamateri et al. (2024) survey the field’s complexities (e.g., technical jargon, diverse classification systems like IPC), reviews key datasets, and evaluates the potential of Deep Learning and large language models. They effectively highlight domain-specific challenges and advancements but concentrate on patent-related methods and data.

For legal text classification, Chalkidis et al. (2019) establish an important benchmark using the EurLex-57K dataset derived from EU legislation. They compare several neural architectures (including CNNs and RNNs), demonstrating that BiGRU-based models generally outperformed CNNs on these legal tasks at the time. This work provides crucial domain-specific insights but focuses on the legal area and architectures predating the current dominance of Transformer models.

*Summary and our contribution*

In summary, while existing surveys and benchmarks offer significant value covering general multi-label classification principles, specific challenges like extreme multi-label learning or label imbalance, or providing deep dives into single domains, a comprehensive, empirical, cross-domain analysis of hierarchical text classification methods has been missing. Our work fills this gap by: (1) surveying methods across multiple domains, (2) proposing a unified framework for comparison, (3) conducting large-scale empirical evaluations on curated textual datasets from five domains, and (4) deriving cross-domain insights and identifying state-of-the-art approaches that transcend their original domain boundaries.

## A Cross-domain Overview of Text Classification with Hierarchical Labels

In this section, we provide the first cross-domain overview of text classification with hierarchical labels. We begin with a formal problem formulation in Sect. 3.1. Then, Sect. 3.2 presents recent methods from different domains. We analyze these methods within our proposed unified framework (Sect. 3.3), focusing on their common submodules (architectural components) and their utilization of label information.

**Table 1:** Overview of analyzed hierarchical text classification methods, showing the method, some key characteristics of the method, and the domain for which the method was designed or on which it was evaluated

Method	Key characteristics	Domain
XR-Transformer 2021	Multi-stage classification with PLT and transformer	Legal/General
MatchXML 2024	Label-text embedding alignment with contrastive learning	Legal/General
X-Transformer 2020	Single-stage classification with PLT and transformer	Legal/General
XR-Linear 2021	Multi-stage classification with PLT and linear classifier	Legal/General
AttentionXML 2019	Label-aware attention with PLT	Legal/General
HILL 2024	Coding tree and contrastive objectives	Sci./News
HiAGM-TP 2020	Structure label encoder and label attention	Sci./News
HiAGM-LA 2020	Holistic structure encoder	Sci./News
HR-SciBERT-mt 2022	Multi-task learning with hierarchical classifiers	Sci
HGCLR 2022	Label-guided contrastive learning	Sci./News
HBGL 2019	Custom attention masking with multi-stage classification	Sci./News
HARNN 2019	Hierarchical attention with recursive neural networks	Patent/Education
LA-HCN 2022	Level-wise label attention and document embedding	Patent/News
PLM-ICD 2022	Segmented text with label-aware attention	Medical
HVHMC 2020	GCN-based inter/intra level dependency modeling	Sci./Patent
HCSM 2021	Multi-component architecture with margin loss	Sci./Legal/News/Patent
FlatBERT 2020	Simple LLM fine-tuning with linear classifier	Patent
THMM 2021	Parent–child representation concatenation	Patent
HTrans 2019	Parent–child parameter sharing	News
Bonsai 2020	Partitioned label trees with multi-stage classification	Legal/General
HyperIM 2020	Hyperbolic embeddings with contrastive learning	News/General
HiLAP 2019	Policy network with negative discounted rewards	News/General
ExMLDS 2019	Scalable label embedding learning	Legal/General
GalaXC 2021	Joint document-label graph with GNN	General
EcLARE 2021	Dynamic inference of label correlation graphs	General
PARL 2024	Model ensemble and learning to rank	Patent
*Plug-in modules*		
CorNet 2020	Correlation-based prediction refinement	Any
DepReg 2023	Dependency regularization for training	Any
PS-PLT 2021	Propensity-scored partitioned label trees	Any
Capsule Net 2019	Label correlation with capsule networks	Any
GANDALF 2024	Graph-based data augmentation for long-tail labels	Any
REMIDIAL 2019	Data augmentation through label correlation	Any

The “General” domain includes methods designed for or evaluated on diverse datasets such as Wikipedia, e-commerce, reviews, web bookmarks, etc., often with a focus on broad applicability rather than domain-specific optimization

### Problem Formulation

Conceptually, text classification with hierarchical labels is the task of assigning one or more relevant labels to a given text input, where these labels are organized in a hierarchical structure. The challenge lies in both understanding the text content and utilizing the relations between labels. The labeling scheme can vary across datasets: some use leaf-only labels (only the most specific labels), some require complete paths (including all ancestors), and some allow partial paths (labels at any level). The prediction task should match the labeling scheme of the dataset.^2^

Formally, the goal of text classification with hierarchical labels is to learn a function $f: X \rightarrow Y^*$, where *X* is the sample space of all possible text sequences and $Y^*$ is the power set of *L* (the label set), so that for any given sample $x \in X, f(x)$ outputs one or more labels most relevant to *x*. The label space can be organized in a hierarchy, defined as a tuple $(L, E, \rho )$ with *L* being a set of labels, $E \subseteq L \times L$ being a set of edges forming a directed acyclic graph (though mostly a tree), and $\rho \in L$ being a unique root node. The hierarchy $(L, E, \rho )$ satisfies: (1) there are no cycles, (2) each node except $\rho$ has at least one parent, and (3) $\rho$ has no parents.

Hierarchical text classification can be viewed as a special case of multi-label classification, where labels are organized in a hierarchical structure rather than being flat. This hierarchical structure distinguishes the task from flat multi-label classification, where labels have no structure to be exploited. The hierarchy can be either given as a taxonomy or learned from the training data via label co-occurrence patterns or other features. The hierarchy provides additional information about label relationships that can be exploited during model training and inference, though the extent and manner of utilizing this information vary across methods.

### Method Selection

We analyzed 32 representative methods published between 2019 and 2024 across multiple domains. While an exhaustive review is beyond our scope, the selected methods encompass diverse approaches and variations, enabling us to identify common patterns and key differences, and to ultimately develop a unified framework. Given our primary goal of establishing this unified architectural framework and conducting a comprehensive empirical comparison of *recent supervised, end-to-end learning* methods, we intentionally focused our selection on this class of approaches. Consequently, this overview prioritizes performance-centric evaluations common in this line of research, and does not delve into unsupervised/semi-supervised methods, purely statistical or symbolic AI approaches, or a detailed analysis of non-performance metrics such as interpretability or computational efficiency, which, while important, represent distinct dimensions of evaluation best explored in dedicated studies.^3^

We started with the recent surveys in Sect. 2 and collected the frequently cited papers, following their citations to find their most related predecessors. We further searched papers published after 2023 from Google Scholar using keywords “hierarchical text classification”, “multi-label text classification”, “patent classification”, “medical coding” and “extreme multi-label classification”. We also included papers that cited the benchmark datasets from Google Scholar and PapersWithCode. To limit the scope, we excluded methods published before 2019, unsupervised methods, and those primarily focused on non-textual datasets. Table 1 provides a concise overview of the 32 methods analyzed in our survey.

**Fig. 1:**
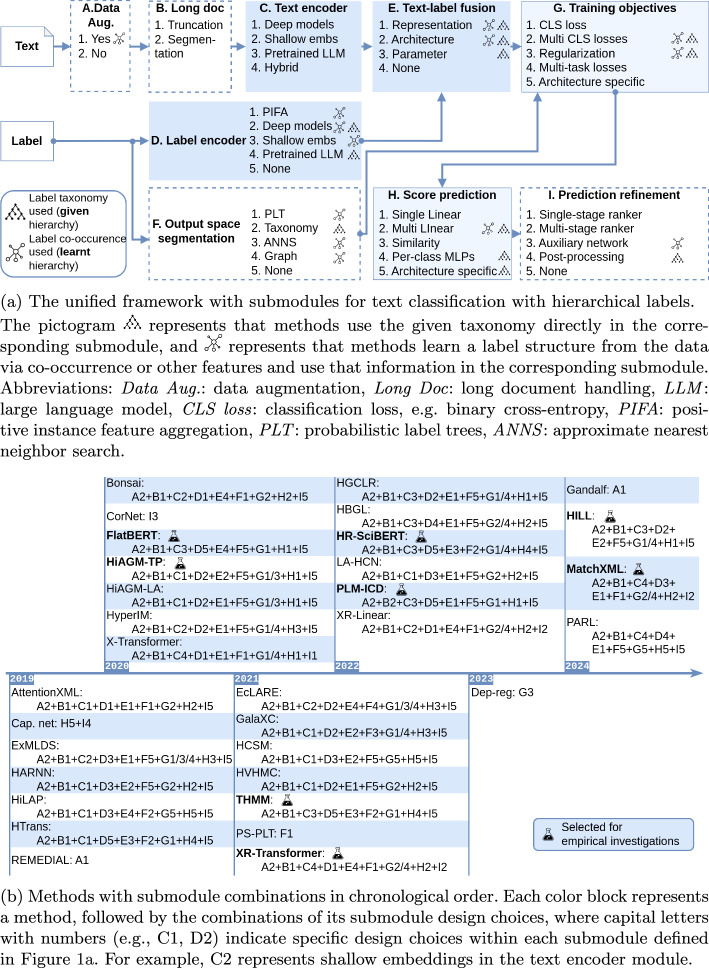
Our framework and methods with submodule combinations

### A Unified Framework of Common Submodules

From the surveyed methods, we abstract a framework constituted by commonly seen architectural components, shown as the nine submodules in Fig. 1a. The framework *begins* with optional preprocessing steps: data augmentation (A) and long document handling (B). The *core processing pipeline* starts with parallel encoding of text and labels: a text encoder (C) transforms input text into embeddings using shallow, deep, or pre-trained models, while a label encoder (D) converts discrete labels into continuous representations, optionally incorporating hierarchical information. These representations are then integrated through text-label information fusion (E), which can occur at representational, architectural, or parameterization levels. For large label spaces, *output space segmentation* (F) may be employed for efficiency. The training process is guided by various training objectives (G), including classification losses and other custom objectives. Finally, the model makes *inferences* through score prediction (H), followed by optional prediction refinement (I) to enhance raw outputs using label correlations or ranking mechanisms.

Utilizing our proposed framework, we locate each method^4^ by its specific instantiations of the submodules in the framework as shown in Fig. 1b. We further emphasize that label hierarchy in the current problem setting can refer to either a given taxonomy or a learned structure, meaning that its usage can be either explicit (using the given taxonomy directly) or implicit (learning a label structure from the data via co-occurrence or other features). Since not all submodules in existing methods utilize label hierarchy information, we explicitly note such usage both in the following discussion and in Fig. 1a.

We now discuss each submodule in detail, highlighting how different methods implement them and utilize label hierarchy information when applicable.

*A. Data augmentation*

Two methods address data imbalance through label information: REMEDIAL (Charte et al., 2019) balances label distribution by decoupling frequent and rare label co-occurrences: it clones high-concurrence instances and edits them so that one version contains only the majority labels and the other only the minority ones, with the guide of the SCUMBLE metric (Charte et al., 2014); while Gandalf (Kharbanda et al., 2024) leverages label co-occurrence graphs to generate soft targets as additional training data.

Beyond these specific multi-label techniques, this submodule can also encompass more *general* strategies to address the long-tail distribution of labels and the issue of extremely rare classes (including those with single instances). Such strategies, while not the primary focus of the methods surveyed, are crucial for practical applications and could include the following. *Label Filtering*: Systematically removing labels that fall below a certain frequency threshold. While this simplifies the problem, it alters the original label space and may not be suitable for all applications. *Instance-level Augmentation for Rare Classes*: Techniques like oversampling instances belonging to rare classes (e.g., SMOTE (Chawla et al., 2002)) or generating synthetic data for these classes. *Advanced Sampling Strategies During Training*: Employing sampling techniques that give more weight or attention to rare classes or hard-to-classify instances. A systematic investigation into the integration and impact of these broader augmentation and data balancing strategies within end-to-end HTC models remains an important area for future research.

*Label info utilization*: Current methods primarily utilize label co-occurrence patterns.

*B. Long document handling*

While most methods simply truncate long documents, PLM-ICD (Huang et al., 2022) employs a more sophisticated approach by segmenting text into smaller parts with pre-defined lengths for later information aggregation. This approach is particularly important for medical notes, which can be lengthy and cannot simply be truncated, as critical information might appear later in the document. PLM-ICD first splits the long text into pre-defined equal-sized segments that fit within BERT’s 512 token window, embeds them separately, and then aggregates these embeddings using a dedicated model architecture that preserves information across the entire document.

*Label info utilization*: Current methods do not explicitly use label information here.

*C. Text encoder*

This essential submodule transforms text into embeddings for input. It has evolved from shallow embeddings to deep learning models, and finally to pre-trained LLMs.

**Table:** 

Shallow embeddings	TF-IDF features: Bonsai (Khandagale et al., 2020), ECLARE (Mittal et al., 2021), ExMLDS (Gupta et al., 2019)
	Hyperbolic embeddings: HyperIM (Chen et al., 2020)
Deep learning models	CNN: HiLAP (Mao et al., 2019)
	LSTM: AttentionXML (You et al., 2019), HARNN (Huang et al., 2019), LA-HCN (Zhang et al., 2022); GRU: HTrans (Banerjee et al., 2019)
	Hybrid CNN-RNN: HiAGM (Zhou et al., 2020), HCSM (Wang et al., 2021)
	GCN: HVHMC (Xu et al., 2021)
	Fixed pre-trained embeddings: GalaXC (Saini et al., 2021)
Pre-trained LLMs	BERT-family: HILL (Zhu et al., 2024), PLM-ICD (Huang et al., 2022), PatentBERT (Lee and Hsiang, 2020), THMM (Pujari et al., 2021), HGCLR (Wang et al., 2022), HBGL (Jiang et al., 2022)
Hybrid approaches	LLM + TF-IDF: X-Transformer (Chang et al., 2020), XR-Transformer (Zhang et al., 2021), MatchXML (Ye et al., 2024)
	LLM + BM25/GraphSAGE: PARL (Li et al., 2024)

*Label info utilization*: Label information is not directly used in this module, as we separate text-label fusion into its own submodule

*D. Label encoder*

This submodule transforms discrete labels into embeddings. Methods vary from implicit relationship modeling (PIFA) to explicit structure encoding (GNNs), with LLM-based approaches offering a middle ground through semantic understanding.

**Table:** 

Positive Instance Feature Aggregation	XR-Transformer (Zhang et al., 2021), X-Transformer (Chang et al., 2020), AttentionXML (You et al., 2019), Bonsai (Khandagale et al., 2020)
Shallow embeddings	Label2Vec: MatchXML (Ye et al., 2024), HCSM (Wang et al., 2021), ExMLDS (Gupta et al., 2019)
	Hyperbolic embeddings: HyperIM (Chen et al., 2020)
Deep learning models	Graph neural networks: HiAGM (Zhou et al., 2020), HVHMC (Xu et al., 2021), HGCLR (Wang et al., 2022), GalaXC (Saini et al., 2021), ECLARE (Mittal et al., 2021), HGCLR (Wang et al., 2022)
	Tree-based models: HiAGM (Zhou et al., 2020), HILL (Zhu et al., 2024)
Pre-trained LLMs	Label text encoding: PARL (Li et al., 2024)
	Custom pretraining: HBGL (Jiang et al., 2022)

*Label info utilization*: Label hierarchies are either directly encoded through specialized architectures (GNNs, tree models) or used to guide the pre-training process

*E. Text-Label information fusion*

This submodule integrates text and label information at three distinct levels. While simple concatenation is the most straightforward approach, learnable fusion mechanisms are more popular since they can adapt to different types of label hierarchies.

**Table:** 

Representational level	Simple concatenation: HVHMC (Xu et al., 2021)
	Custom attention: HBGL (Jiang et al., 2022), PLM-ICD (Huang et al., 2022), AttentionXML (You et al., 2019), LA-HCN (Zhang et al., 2022), HiAGM-LA (Zhou et al., 2020)
	Embedding alignment: MatchXML (Ye et al., 2024), PARL (Li et al., 2024), HyperIM (Chen et al., 2020), ExMLDS (Gupta et al., 2019)
	Label-informed positive pairs generation: HGCLR (Wang et al., 2022)
Architectural level	Graph-based: HiAGM-TP (Zhou et al., 2020), GalaXC (Saini et al., 2021)
	RNN-based: HiAGM-TP (Zhou et al., 2020), HARNN (Huang et al., 2019), HILL (Zhu et al., 2024), HCSM (Wang et al., 2021)
	Capsule networks: HCSM (Wang et al., 2021)
Parameterization level	Hierarchy-aware parameters sharing or initialization: THMM (Pujari et al., 2021), HR-SciBERT-mt (Sadat and Caragea, 2022), HTrans (Banerjee et al., 2019)

*Label info utilization*: Hierarchical label information is used to guide attention mechanisms, embedding alignments, and parameter settings, enhancing the fusion of text and label data

*F. Output space segmentation*

This submodule restructures the prediction space, crucial for reducing huge label sets.

**Table:** 

PLT^1^	XR-Transformer (Zhang et al., 2021), MatchXML (Ye et al., 2024), X-Transformer (Chang et al., 2020), AttentionXML (You et al., 2019), Bonsai (Khandagale et al., 2020)
ANNS^2^	GalaXC (Saini et al., 2021)
Taxonomy^3^	THMM (Pujari et al., 2021), HTrans (Banerjee et al., 2019), HiLAP (Mao et al., 2019)
Graph-based	ECLARE (Mittal et al., 2021)

^1^PLT: Probabilistic Label Trees partition the label space into a tree structure for efficient prediction

^2^ANNS: Approximate Nearest Neighbor Search uses similarity search to reduce the candidate label space

^3^Taxonomy: Direct usage of the given hierarchical structure for label space segmentation

*Label info utilization*: Hierarchical label structures are leveraged to segment the output space, improving efficiency and scalability in large label environments.

*G. Training objectives*

This submodule involves various loss functions and training strategies, from simple classification losses to sophisticated approaches that capture both semantic relationships and hierarchical structure.

**Table:** 

Classification loss	Binary cross entropy: basic building blocks of most methods
Multiple classification losses	Multi-stage: XR-Transformer (Zhang et al., 2021), MatchXML (Ye et al., 2024), X-Transformer (Chang et al., 2020), AttentionXML (You et al., 2019), Bonsai (Khandagale et al., 2020)
	Multi-level: HVHMC (Xu et al., 2021), HARNN (Huang et al., 2019), LA-HCN (Zhang et al., 2022), HBGL (Jiang et al., 2022)
Regularization	Hierarchical constraints: HiAGM (Zhou et al., 2020)
	Label co-occurrence: ExMLDS (Gupta et al., 2019)
Multi-task learning	Label-prediction pretraining: HBGL (Jiang et al., 2022)
	Keyword prediction: HR-SciBERT-mt (Sadat and Caragea, 2022)
	Contrastive learning: XR-Transformer (Zhang et al., 2021), MatchXML (Ye et al., 2024), X-Transformer (Chang et al., 2020)
	Embedding refinement: MatchXML (Ye et al., 2024), HILL (Zhu et al., 2024), HGCLR (Wang et al., 2022), ExMLDS (Gupta et al., 2019), GalaXC (Saini et al., 2021), ECLARE (Mittal et al., 2021)
Architecture-specific	Reinforcement learning rewards: HiLAP (Mao et al., 2019)
	Capsule network margin loss: HCSM (Wang et al., 2021)
	Ranking loss: PARL (Li et al., 2024)

*Label info utilization*: Label hierarchical information guides both loss function design and regularization strategies, particularly in multi-stage and multi-task approaches

*H. Score Prediction*

This submodule transforms model outputs into label predictions, showing different approaches to the task: basic linear layers treat each label as an independent binary decision; approaches with multiple classifiers each handle labels differently to capture their relationships, while similarity-based methods exploit learned embedding spaces.

**Table:** 

Linear	Basic approach: a single linear layer followed by optional sigmoid activation.
Multiple Linear	Multi-stage: XR-Transformer (Zhang et al., 2021), MatchXML (Ye et al., 2024), X-Transformer (Chang et al., 2020), AttentionXML (You et al., 2019)
	Multi-resolution: HVHMC (Xu et al., 2021), HARNN (Huang et al., 2019), LA-HCN (Zhang et al., 2022), HBGL (Jiang et al., 2022)
Similarity-based	HyperIM (Chen et al., 2020), ExMLDS (Gupta et al., 2019), GalaXC (Saini et al., 2021), ECLARE (Mittal et al., 2021)
Per-class MLPs	A separate network for each label: THMM (Pujari et al., 2021), HR-SciBERT-mt (Sadat and Caragea, 2022)
Architecture-specific	Capsule networks: HCSM (Wang et al., 2021)
	Policy networks: HiLAP (Mao et al., 2019)
	Learning-to-rank: PARL (Li et al., 2024)

*Label info utilization*: Label hierarchical information influences prediction strategies through multi-stage architectures, label-specific networks, and specialized prediction mechanisms

**Table:** 

Single-stage ranking	X-Transformer (Chang et al., 2020)
Multi-stage ranking	Hierarchical rankers: XR-Transformer (Zhang et al., 2021), MatchXML (Ye et al., 2024), X-Transformer (Chang et al., 2020)
Auxiliary networks	After-prediction network to utilize label relationships: CorNet (Xun et al., 2020)
Post-processing	Data-specific refinement: HCSM (Wang et al., 2021)

*Label info utilization*: While CorNet and post-processing rules explicitly leverage label structure, ranking-based approaches focus primarily on computational efficiency for large label spaces rather than hierarchical relationships

Each method discussed in this paper can be characterized by whether and how it instantiates each submodule in the framework, as illustrated chronologically in Fig. 1b. While methods may utilize the same types of submodules, their specific choices and implementations within each submodule can vary significantly, with detailed comparisons provided in Table 11.

## Cross-domain Evaluation

We conduct a comprehensive cross-domain evaluation to reveal the true generalizability of hierarchical text classification methods. We first explain our methodology and rationale for this benchmark (Sect. 4.1), then select representative datasets (Sect. 4.2) and methods (Sect. 4.3) from different domains. We evaluate each method’s performance across all datasets using precision and recall metrics (Sect. 4.5). Section 4.6 discusses the general trends and observations emerging from this analysis.

### Methodology and Rationale

Existing HTC benchmarks are typically “domain-isolated”: medical methods are evaluated solely on MIMIC datasets, while patent classification methods are tested on USPTO datasets. This prevents us from understanding whether a method’s success reflects universally effective architecture or domain-specific optimization. Our cross-domain benchmark distinguishes generalizable innovations from domain overfitting.

#### Why Existing Benchmarks Are Inadequate

Current benchmarks have three critical limitations: (1) **Inconsistent evaluation protocols**: some papers report Micro-F1 while others report Precision@k, with different preprocessing; (2) **No difficulty variation**: methods are not tested across diverse document lengths, hierarchy depths, or label sparsity patterns; (3) **Hidden failure modes**: a method optimized for short WOS texts may fail on long MIMIC documents, but this remains invisible in single-domain evaluation.

We therefore constructed a unified benchmark with: (1) eight datasets across five domains with standardized preprocessing; (2) consistent metrics (Precision@k, Recall@k) across all method-dataset pairs; (3) systematic stress-testing of methods against characteristics they were not designed for. This isolates architectural effectiveness from domain-specific tuning.

Consequently, we constructed a completely new benchmark suite that: (1) aggregates eight datasets across five distinct domains with unified preprocessing; (2) applies consistent evaluation metrics (Precision@k, Recall@k) across all method-dataset combinations; and (3) ensures that every method is stress-tested against data characteristics (detailed in Sect. 4.2) it was never originally designed to handle. This design allows us to isolate the effect of architectural choices from domain-specific tuning.

#### Adaptation Strategy: Interface Wrappers

Leveraging the Unified Framework proposed in Sect. 3, we evaluate most methods using their original codebases with minimal modifications. We do not alter the internal architectures or loss functions (the “brain” of the method). Instead, we standardize the Input (Text Encoder) and Output (Prediction Head) interfaces. For example, to apply a method designed for flat labels to a hierarchical dataset, we map the hierarchy to the method’s expected input format without changing how the method learns features. This “lightweight extension” strategy allows us to evaluate the robustness of the core method. While some methods required framework adaptation (e.g., TensorFlow to PyTorch), the core architectures and learning procedures remained unchanged.

Regarding label rarity, rather than modifying original methods to artificially handle unbalanced hierarchies, we employ standard metrics (Precision@k and Recall@k) across all datasets. This allows us to treat label imbalance as a dataset variable to be analyzed, objectively measuring how well each original architecture copes with the long-tail distributions inherent in real-world cross-domain scenarios.

### Datasets

Having established our methodology, we now introduce the eight datasets used in our cross-domain benchmark. We characterize each dataset along multiple dimensions and detail the data cleaning and preprocessing procedures to ensure fair comparisons.

#### Selection

We consider datasets from five domains for cross-domain evaluation: legal, scientific, news, medical, and patent classification. We selected these domains to represent a diverse set of common and well-established application areas where hierarchical text classification is frequently employed, as evidenced by their regular appearance in relevant surveys and benchmark studies. Our selection criteria focused on datasets meeting several key requirements for a rigorous cross-domain HTC analysis:

1. **Gold-standard taxonomies** Availability of a defined, hierarchical label structure is essential for evaluating HTC methods.
2. **Textual content** Availability of the original document text is crucial for applying and evaluating modern NLP models (unlike datasets providing only pre-processed features).
3. **Significant Hierarchical Complexity** Our study prioritizes datasets where the label hierarchy is sufficiently complex to robustly test and differentiate methods specifically designed to leverage such structures. This typically, though not exclusively, correlates with a larger number of interconnected labels. Evaluating specialized HTC methods on datasets with very few labels might not fully reveal their unique advantages or limitations compared to simpler flat classification approaches.
4. **Manageable label spaces** While covering a range, we focused on datasets with fewer than 4,000 labels to maintain a practical scope for evaluation across multiple methods, differentiating from extreme multi-label classification scenarios that might necessitate entirely different techniques.
5. **Varied characteristics** Inclusion of datasets spanning a significant range of sizes (from tens of thousands to millions of samples), document lengths, and label cardinalities to assess method robustness.

These criteria led to the selection of eight representative datasets: EurLex-3985, EurLex-DC-410, WOS-141, NYT-166, SciHTC-83, SciHTC-800, MIMIC3-3681, and USPTO2M-632. The naming format of the datasets is Dataset-N, where N is the **number of labels**. It is important to note that while all selected datasets happen to have over 80 labels, this number was an outcome of prioritizing hierarchical complexity for evaluating specialized HTC methods, rather than a strict exclusionary threshold for datasets with fewer labels.

#### Characteristics of Datasets

To facilitate systematic comparison, we characterize each dataset along multiple dimensions, detailed in Table 2. These include: basic properties like domain and document length statistics; label space characteristics such as the total number of labels (“#Labels”), the number of unique label combinations (“Distinct Label Sets”), and the maximum hierarchy depth (“Max Depth”); label assignment patterns reflected in the statistics for the number of labels per sample (“Avg/Max/Min/Std #Labels per sample”); and label distribution and diversity metrics, including statistics on the number of samples per label (“Avg/Max/Min/Std #Samples per label”) and the average rarity of label combinations (“Avg Pattern IDF”). These metrics offer a nuanced view of each dataset’s complexity and distribution, which can influence model performance.

**Table 2:** Summary of datasets

Dataset	Domain	Avg words	#Lbls	Max depth	Dist. sets	Avg. IDF	Lbls/Samp (A/Max/Min/Std)	Samps/Lbl (A/Max/Min/Std)	Train+Dev/ Test
EurLex-3956	Legal	2635	3956	2	16,467	6.8	5.3/24/1/1.4	26.0/1253/1/68.3	15,449/3865
EurLex-3985*	Legal	2635	3985	2	16,434	6.8	12.8/38/3/3.2	62.0/8479/1/348.9	15,444/3862
EurLex-DC-410*	Legal	2635	410	2	1615	6.1	1.3/7/1/0.6	61.0/1909/1/152.1	15,472/3868
WOS-141	Sci	200	141	2	134	1.9	2/2/2/0	666.5/14,625/1/1585.8	37,588/9397
NYT-166	News	606	166	8	8288	6.6	7.6/38/1/5.6	1665.1/24,554/143/3444.7	29,209/7262
SciHTC-83	Sci	145	83	6	83	0.5	1.8/2/1/0.4	4091.2/32,854/156/6586.8	167,544/18,616
SciHTC-800*	Sci	145	800	6	798	3.3	1.6/2/1/0.5	369.8/17,166/5/1184.1	167,544/18,616
MIMIC3-3681	Med	1514	3681	0 (+ 3)	52,453	6.9	15.6/65/1/8.0	223.2/20,046/10/819.4	43,978/8734
USPTO2M-632	Patent	117	632	0 (+ 2)	71,841	6.5	1.3/18/1/0.7	4239.6/281,876/1/14688.3	1,948,508/49,900
USPTO10k-632**	Patent	116	632	0 (+ 2)	13,191	6.6	1.9/18/1/1.1	178.8/10,433/1/577.0	10,000/49,900
USPTO100k-632**	Patent	116	632	0 (+ 2)	23211	6.6	1.8/18/1/1.1	418.8/23895/1/1313.6	100000/49900

Avg = Average; Lbls = Labels; Dist. = Distinct; Samp = Sample; A = Average; Dist. Sets = Number of distinct label combinations in the dataset. *New variant of the dataset introduced for this study

**Subset created for ablation studies. It shows the number of unique label combinations (patterns) in the dataset

Avg. IDF = Avg Pattern IDF. It measures the average Inverse Document Frequency of these patterns, where higher values indicate greater diversity and rarity of label combinations (calculated as $\frac{1}{|\mathcal {P}|} \sum _{p \in \mathcal {P}} \log \frac{N}{f_p+1}$, where $\mathcal {P}$is the set of unique patterns, *N* is the total number of samples, and $f_p$ is the frequency of pattern *p*)

(+*n*) in Max Depth indicates that *n* additional levels were added to the originally flat label annotation

#### Data Preparation and Partitioning

While we maintain consistency with established preprocessing procedures where possible, several datasets required specific handling to ensure compatibility with our evaluation framework, and we also created new versions of some datasets for evaluation.

1. EurLex-3956/3985: Introduced by Loza Mencía and Fürnkranz (2008) and known as EurLex-4k (Bhatia et al., 2016) in the literature; it contains European legal documents sourced from the EUR-Lex repository.^5^
  - Label processing: The original dataset is annotated with 3956 labels of EUROVOC descriptors, describing a wide range of EU-related topics. But the labels are raw text and not mapped to any existing taxonomy. We mapped them to the EUROVOC taxonomy^6^ using an ensemble of string similarity metrics^7^ and manual verification, resulting in 3890 mapped labels. We then enriched the labels by adding parent codes, yielding a final label set of size 3985. We maintain both versions: EurLex-3956 for literature comparison and EurLex-3985 as our cleaned version for evaluation.
  - Text processing: We extracted the original text from HTML sources^8^ rather than using the previously tokenized version from the AttentionXML repository^9^ or BOW features from the repository maintained by Bhatia et al. (2016), enabling optimal tokenization for different pretrained language models.
2. EurLex-DC-410: Using the original EurLex corpus, we created a new dataset annotated with the Directory Codes (DC). These codes represent classes used in the Directory of Community Legislation in Force. The hierarchy consists of 20 top-level chapter headings with up to four levels of subdivisions. After filtering documents to retain only those with valid DC annotations, the final dataset contains 410 labels.
3. WOS-141: Introduced by Kowsari et al. (2017), it contains scientific article abstracts from the Web of Science. The labels represent a hierarchical taxonomy of scientific categories and subcategories, with a total number of 141. We obtained the data from the original source.^10^
4. NYT-166: Originating from the New York Times Annotated Corpus (Sandhaus, 2008), this dataset consists of news articles spanning diverse topics. The 166 labels form a hierarchical taxonomy representing thematic categories from general subjects down to more fine-grained topics.^11^
5. SciHTC-83: Introduced by Sadat and Caragea (2022), this dataset comprises scientific abstracts from multiple research fields. The 83 labels represent a selection of the most frequently occurring subject categories, structured hierarchically to capture broad domains down to more specialized subfields. We use the same processed version provided by Sadat and Caragea (2022).
6. SciHTC-800: We build SciHTC-800 from the original SciHTC dataset by adding the second-level codes and correcting the inconsistent codes, resulting in a dataset with 800 labels.
7. MIMIC3-3681: The MIMIC-III clinical database (Johnson et al., 2016) consists of de-identified hospital discharge summaries annotated with International Classification of Diseases (ICD) codes. These codes span broad medical domains, covering both diagnoses and procedures. We use the MIMIC3-clean variant introduced by Edin et al. (2023), which provides a refined version of the dataset optimized for clinical classification tasks.
8. USPTO2M-632: Introduced by Li et al. (2018), this dataset is derived from United States Patent and Trademark Office (USPTO) documents. It contains patent abstracts annotated with hierarchical patent classification codes, reflecting technological fields and subfields. We obtained the data from the original source.^12^

For experimental partitioning, we adhered to established train/test splits wherever available to ensure comparability with prior work. Specifically:

1. For MIMIC3-3681, we used the official splits provided with the MIMIC3-clean version provided by Edin et al. (2023). This version was specifically created to ensure most codes are present in both training and test sets, employing multi-label stratified sampling for document assignment, and ensuring patient-level disjointness between splits.
2. For WOS-141 and NYT-166, we followed the random splits used in the study by Zhu et al. (2024).
3. For SciHTC-83 and our derived SciHTC-800, we used the original random splits provided by Sadat and Caragea (2022).
4. For USPTO2M-632 (and its subsets), we adopted the standard chronological split commonly used in patent classification benchmarks, e.g., Lee and Hsiang (2020), which is not label-stratified.
5. For EurLex-3956 (original version), we performed a random split following the approach used in the widely cited XML repository (Bhatia et al., 2016). For the two derived datasets EurLex-3985 and EurLex-DC-410, we performed a random split as the original EurLex-3956.

While stratified sampling, such as Iterative Stratification for multi-label data (Sechidis et al., 2011), is often advocated for problems with many classes and high class imbalances, there are several arguments against it in our current study. First, for most of the datasets, non-stratified benchmark splits are already established, such that using a new stratified partitioning would harm comparability with prior work (Bhatia et al., 2016; Zhu et al., 2024; Lee and Hsiang, 2020; Edin et al., 2023). Moreover, hierarchical stratification that preserves label distributions across all hierarchy levels remains conceptually challenging and is an open research problem. Additionally, general multi-label stratification methods face computational challenges with larger datasets (Merrillees and Du, 2021). Finally, stratified sampling may provide over-optimistic performance estimates compared to random splits in situations where the label distribution in the dataset differs substantially from the label distribution in the wild. Our exploratory experiment on EurLex-3956 (detailed in “Appendix 8.5”) does indeed confirm that stratified splitting leads to a small increase in the performance measures. For these reasons, we decided to use the established splits for the existing datasets, and to use a similar splitting strategy for the two newly curated datasets so as to ensure consistency across the study.

It is important to acknowledge that for datasets where such specific preprocessing (like frequency-based label filtering and multi-label stratification as seen in MIMIC3-clean) was not part of the established split, they exhibit long-tail label distributions where some classes may have very few instances (Table 2). For these datasets, in line with common practice for broad comparative studies and to ensure a consistent baseline, no explicit mechanism beyond ensuring the model’s output layer accommodates all unique classes was implemented. This ensures all models in our comparative study face the same potential challenge regarding these extremely rare classes on those specific datasets. We recognize this means some single-instance classes might not appear in a given split, which could affect performance assessment for those specific classes; this aspect is further discussed in our evaluation metrics (Sect. 4.4).

*Data adaptation for methods across domains* Since all datasets have given taxonomies, we handle them differently based on each method’s requirements:

1. For methods that utilize the given taxonomies (e.g., THMM, HILL, HiAGM), we follow their specific preprocessing procedures to incorporate the taxonomies.
2. For methods that don’t use the given taxonomies (e.g., XR-Transformer), we simply preprocess the text according to their requirements while disregarding the taxonomies.

Two datasets required special handling due to their flat label annotation structures. MIMIC3-3681 contains only the most specific diagnostic and procedure codes (3681 flat labels), so we expanded its hierarchy by including upper-level codes to create a three-level structure compatible with THMM/HILL/HiAGM. Similarly, USPTO2M-632 contains only subclass labels (632 flat labels), so we added parent and grandparent labels to create a two-level hierarchy. For fair comparison, evaluations on both datasets were still conducted using only the original flat labels.

### Methods

We carefully select representative methods for cross-domain evaluation, focusing on those that effectively utilize or can be adapted to use label hierarchies. Our selection process balances comprehensive coverage with practical resource constraints.

#### Selection

Resource limitations prevent us from experimenting with all 32 methods surveyed above; therefore, we select representative methods based on three criteria:

1. Performance: We prioritize methods that achieved state-of-the-art results on their respective datasets, identified through recent surveys (Sect. 2) and 2024 publications.
2. Label hierarchy utilization: We focus on methods that either integrate label hierarchical information into their main architecture or can be readily adapted to do so, excluding augmentation or post-processing components. An exception is made for PatentBERT/FlatBERT, which serves as a baseline model without label hierarchy information used anywhere.
3. Implementation adaptability: For reproducibility and extensibility, we prioritize methods with clear architectures that are easy to implement and modify. We exclude ensemble methods to maintain architectural clarity, facilitate potential improvements, and manage computational resources.

#### Selected Methods for Benchmark

Based on these criteria, we selected eight representative methods covering five domains:

*Legal Domain* The current state-of-the-art on EurLex-3956, **MatchXML** (2024), extends XR-Transformer by incorporating advanced label encoding mechanisms. Its predecessor, **XR-Transformer** (2021), remains a widely cited baseline for hierarchical text classification.^13^

*Scientific and News Domains* In these domains, **HILL** (2024) advances the state-of-the-art on both WOS-141 and NYT-166 through novel hierarchy learning, text-label fusion mechanisms, and contrastive learning objectives. Its predecessor, **HiAGM-TP** (2020), established the foundation for graph-based label hierarchy modeling. **HR-SciBERT-mt** (2022) achieves state-of-the-art performance on SciHTC-83, a dataset introduced alongside the model, through multi-task learning and hierarchical classifiers.

*Medical Domain* For medical text classification, **PLM-ICD** (2022) achieves state-of-the-art performance on MIMIC3-3681 by addressing long document handling and incorporating label-aware attention mechanisms.

*Patent Domain* In patent classification, PatentBERT/**FlatBERT** (2020) demonstrates that fine-tuning pretrained language models, such as BERT, with a simple classification head can achieve state-of-the-art performance on USPTO2M-632. **THMM** (2021) takes a different approach, achieving state-of-the-art results on its own sampled dataset USPTO70k through hierarchical classifier architectures.

Note that some methods face computational and architectural **constraints**. HiAGM-TP and HILL require complete label hierarchies and substantial memory resources for large label spaces. THMM encounters similar limitations in both hierarchical structure requirements and memory usage. HR-SciBERT-mt presents additional requirements, needing keyword annotations and significant computational resources, particularly for large-scale datasets.

### Evaluation Metrics

We primarily focus on precision@*k* and recall@*k* in our analysis as they directly measure the accuracy of the top predictions and the retrieval effectiveness for relevant labels, which are key aspects of evaluating multi-label classification systems. For a given instance, precision@*k* is the number of true positive labels in the top-*k* predictions divided by *k*, while recall@*k* is the number of true positive labels in the top-*k* predictions divided by the total number of true labels for that instance. These metrics are then averaged across all test instances. We also report the ranks of the methods on each dataset.

While some applications, particularly in domains like automated medical coding (e.g., Edin et al. (2023)), may benefit from converting ranked predictions into a definite set of labels using a tuned threshold, the majority of recent research in hierarchical and extreme multi-label classification, including most of the methods surveyed in this paper, evaluates performance based on top-*k* ranked outputs. Adopting this top-*k* evaluation approach allows for a more direct and unified comparison across these diverse methods. Furthermore, determining optimal thresholds typically requires additional per-dataset and per-model tuning, which would introduce significant complexity into a broad comparative study like the current one. Therefore, our evaluation centers on rank-aware metrics.

F1-scores provide another valuable perspective, particularly concerning class imbalance, and we present them in the “Appendix” for completeness (see Table 18). These F1 scores were computed based on the top-10 predictions for each instance. Consequently, Micro-F1 trends largely align with the P/R@10 results already presented in the main text. Macro-F1 scores, however, exhibited high variance across experimental runs; this variability likely arises from the amplification of prediction inconsistencies for rare labels (which are common in these datasets with large label spaces). This instability limits their reliability for consistent cross-domain comparisons, and therefore, these F1 scores are not the focus of our main textual analysis.

It is important to note that when evaluating with these standard metrics on datasets with extremely rare classes, the performance on such classes can be volatile or difficult to capture. While some domain-specific evaluations employ metrics adjusted for label rarity (e.g., propensity-scored metrics), for this broad cross-domain comparison, we adhered to widely used precision@k, recall@k, and F1 scores to maintain simplicity and comparability across diverse methods.

### Results

We evaluate each selected method on all eight datasets using the metrics defined in Sect. 4.4. While we report results for various values of *k* ($k=1, 3, 5, 8, 10$), our primary discussion will often focus on precision@1 and recall@1 (Tables 3 and 4). This emphasis reflects the importance in many applications of correctly identifying the single most relevant label and provides a sensitive measure for comparing top-performing models. However, we strongly encourage readers to also consider the results at $k=10$ (Tables 5 and 6), as they offer crucial insights into the models’ ability to retrieve a broader set of correct labels. Results for $k=3, 5, 8$ are provided in “Appendix 8”.

General trends and high-level observations are discussed in the following Sect. 4.6, while in-depth analysis is provided later in Sect. 5.

All experiments were conducted using PyTorch (Paszke et al., 2019) on a single NVIDIA A40 GPU, including our re-implementations of FlatBERT and THMM. We maintained consistent dataset processing and train/test splits across all methods while adhering to each method’s specific requirements. For each method-dataset combination, we conducted hyperparameter tuning using a validation set randomly sampled from the training data, with limited search ranges closely following the original papers. All reported results are averages across five random seeds.

To manage computational resources, we established uniform constraints for each experimental run (defined as training one method on one dataset with one random seed using the default epoch count from the original papers). These constraints include a maximum GPU memory usage of 40GB and a time limit of 36 h. Experiments exceeding these limits are denoted as “EM” (exceeded memory) or “ET” (exceeded time) in our results.^14^

*Caveat* While we carefully reproduced all methods following their original implementations and validated against reported results where possible, our evaluation setup may differ from the original papers. For methods reporting the same metrics, our reproduced results closely match published numbers (see the following paragraph on reproduced results). However, many papers use different evaluation metrics, making direct comparisons impossible. Therefore, the performance numbers reported in this section reflect results under our unified evaluation framework, which enables fair comparisons but may not exactly match previously published results.

**Table 3:** Cross-domain Precision@1 scores and ranks

Method	WOS -141	NYT -166	EurLex -3985	EurLex-DC -410	SciHTC -83	SciHTC -800	MIMIC3 -3681	USPTO2M -632
MatchXML	86.30 - **6**	96.27 - **2**	97.26 - **2**	89.88 - **1**	61.70 - **1**	41.28 - **1**	86.33 - **3**	82.28 - **1**
XR-Transformer	86.49 - **5**	96.64 - **1**	97.51 - **1**	89.77 - **2**	61.53 - **3**	40.69 - **2**	86.88 - **2**	80.99 - **4**
HILL	90.30 - **1**	95.02 - **4**	94.59 - **4**	86.12 - **4**	57.70 - **7**	35.04 - **7**	80.12 - **4**	ET
HiAGM-TP	89.75 - **3**	89.44 - **7**	90.76 - **5**	85.19 - **5**	57.68 - **8**	37.57 - **6**	EM	ET
PLM-ICD	89.88 - **2**	96.03 - **3**	94.77 - **3**	87.36 - **3**	61.40 - **4**	38.40 - **5**	90.77 - **1**	80.73 - **5**
FlatBERT	82.65 - **7**	90.95 - **6**	44.43 - **7**	10.08 - **7**	60.45 - **5**	38.63 - **4**	25.84 - **5**	81.36 - **3**
THMM	89.04 - **4**	93.84 - **5**	69.94 - **6**	49.74 - **6**	61.64 - **2**	39.17 - **3**	EM	81.83 - **2**
HR-SciBERT-mt	ET	ET	ET	ET	59.46 - **6**	ET	ET	ET

ET the method exceeded the time limit, EM the method exceeded the memory limit. Same for the following tables

**Table 4:** Cross-domain Recall@1 scores and ranks

Method	WOS -141	NYT -166	EurLex -3985	EurLex-DC -410	SciHTC -83	SciHTC -800	MIMIC3 -3681	USPTO2M -632
MatchXML	43.15 - **6**	22.78 - **2**	8.06 - **2**	78.89 - **1**	36.33 - **2**	27.84 - **1**	7.17 - **3**	55.53 - **1**
XR-Transformer	43.25 - **5**	22.93 - **1**	8.09 - **1**	78.88 - **2**	36.29 - **3**	27.43 - **2**	7.22 - **2**	54.75 - **4**
HILL	45.15 - **1**	22.45 - **4**	7.80 - **4**	75.64 - **4**	33.89 - **7**	23.48 - **7**	6.59 - **4**	ET
HiAGM-TP	44.88 - **3**	21.21 - **7**	7.49 - **5**	74.91 - **5**	33.80 - **8**	24.77 - **6**	EM	ET
PLM-ICD	44.94 - **2**	22.76 - **3**	7.84 - **3**	76.64 - **3**	36.28 - **4**	25.24 - **5**	7.63 - **1**	54.45 - **5**
FlatBERT	41.33 - **7**	21.45 - **6**	3.31 - **7**	9.30 - **7**	35.78 - **5**	25.82 - **3**	1.76 - **5**	54.92 - **3**
THMM	44.52 - **4**	22.09 - **5**	5.68 - **6**	44.77 - **6**	36.37 - **1**	25.56 - **4**	EM	55.26 - **2**
HR-SciBERT-mt	ET	ET	ET	ET	34.63 - **6**	ET	ET	ET

**Table 5:** Cross-domain Precision@10 scores and ranks

Method	WOS -141	NYT -166	EurLex -3985	EurLexDC -410	SciHTC -83	SciHTC -800	MIMIC3 -3681	USPTO2M -632
MatchXML	18.86 - **5**	54.61 - **2**	78.01 - **2**	12.10 - **3**	15.75 - **5**	10.98 - **3**	58.77 - **3**	16.21 - **4**
XR-Transformer	19.01 - **4**	55.01 - **1**	78.14 - **1**	12.17 - **2**	15.43 - **7**	10.58 - **5**	58.92 - **2**	15.55 - **5**
HILL	19.31 - **1**	52.40 - **4**	77.13 - **3**	12.25 - **1**	14.89 - **8**	9.84 - **7**	50.25 - **4**	ET
HiAGM-TP	19.20 - **2**	45.79 - **7**	70.91 - **5**	11.98 - **5**	15.60 - **6**	10.58 - **5**	EM	ET
PLM-ICD	19.17 - **3**	54.55 - **3**	73.61 - **4**	12.01 - **4**	16.21 - **1**	10.99 - **2**	67.11 - **1**	16.94 - **3**
FlatBERT	14.54 - **7**	48.22 - **6**	28.70 - **7**	2.20 - **7**	15.95 - **4**	10.71 - **4**	7.19 - **5**	17.08 - **2**
THMM	18.63 - **6**	52.08 - **5**	35.56 - **6**	8.52 - **6**	16.14 - **2**	11.06 - **1**	EM	17.16 -**1**
HR-SciBERT-mt	ET	ET	ET	ET	16.14 - **2**	ET	ET	ET

**Table 6:** Cross-domain Recall@10 scores and ranks

Method	WOS -141	NYT -166	EurLex -3985	EurLexDC -410	SciHTC -83	SciHTC -800	MIMIC3 -3681	USPTO2M -632
MatchXML	94.29 - **5**	83.87 - **2**	62.67 - **2**	95.74 - **3**	86.62 - **5**	70.39 - **3**	43.90 - **3**	89.34 - **4**
XR-Transformer	95.05 - **4**	84.38 - **1**	62.79 - **1**	96.34 - **2**	84.91 - **7**	67.64 - **6**	43.99 - **2**	86.68 - **5**
HILL	96.53 - **1**	79.70 - **5**	61.91 - **3**	96.72 - **1**	82.17 - **8**	63.50 - **7**	37.26 - **4**	ET
HiAGM-TP	96.01 - **2**	71.85 - **7**	56.67 - **5**	95.10 - **4**	85.78 - **6**	68.27 - **5**	EM	ET
PLM-ICD	95.84 - **3**	83.58 - **3**	59.03 - **4**	95.07 - **5**	89.23 - **1**	70.82 - **2**	50.25 - **1**	92.13 - **3**
FlatBERT	72.70 - **7**	72.93 - **6**	22.13 - **7**	19.99 - **7**	87.89 - **4**	69.73 - **4**	5.19 - **5**	92.61 - **2**
THMM	93.13 - **6**	80.65 - **4**	27.56 - **6**	69.44 - **6**	88.81 - **3**	70.99 - **1**	EM	92.90 - **1**
HR-SciBERT-mt	ET	ET	ET	ET	88.83 - **2**	ET	ET	ET

*Results not included in main comparisons* For literature comparison, we validated our implementations against the original EurLex-3956 dataset (also known as EurLex-4k) before using our cleaned version EurLex-3985. Our reproduced precision@1/recall@1 scores (MatchXML: 87.89/17.84, XR-Transformer: 87.83/17.82) closely match reported results (88.12/-, 87.22/- respectively), confirming implementation fidelity.

Analysis of precision and recall scores at higher *k* values reveals expected precision-recall trade-offs across all datasets: precision decreases while recall increases as *k* grows. The magnitude of this trade-off varies by dataset: WOS shows dramatic precision drops ($\sim$70 percentage points from $k=1$ to $k=10$), while MIMIC3-3681 exhibits more gradual degradation ($\sim$ 25 points). Notably, most model rankings remain stable. A more detailed analysis and the numerical results are provided in “Appendix 8.1”.

### General Observations

**Fig. 2:**
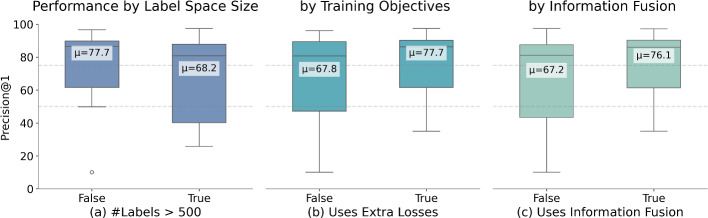
General trends in model performance across different datasets. **a** Label space complexity: Performance decreases with larger label sizes. **b** Model architecture: LLM-based models with advanced learning strategies outperform simpler architectures. **c** Text-label information fusion: Sophisticated mechanisms for combining text and label information yield better results than basic approaches

Here, we report our high-level observations based on the main cross-domain results, focusing on patterns and insights that emerge from evaluating the methods in their original proposed forms. In the following Sect. 5, we will conduct more systematic analyses and ablation studies to examine the effects of specific design choices and further dissect the observed trends. Figure 2 visualizes three main trends:

*Label space complexity* strongly influences model performance, as shown in Fig. 2a. Methods achieve higher scores on datasets with fewer labels (under 500), while performance declines as label count increases. Additionally, as seen in Tables 2 and 4, high per-sample label cardinality (MIMIC3-3681 with 15.6 labels/sample and EurLex-3985 with 12.8 labels/sample) presents challenges, with methods generally achieving lower recall scores on such datasets.

*Model architecture* plays a crucial role, as illustrated in Fig. 2b. Models using large language models as text encoders consistently outperform others, particularly when incorporating advanced learning strategies such as contrastive losses or regularization terms in their training objectives.

*Text-label interaction mechanisms* prove important, demonstrated in Fig. 2c. Methods that effectively combine text and label information through sophisticated mechanisms, such as embedding alignment or label-aware attention, show marked improvements over simpler approaches.

Beyond these salient general trends, some more nuanced patterns emerge across datasets and methods, which are presented in “Appendix 8.3”.

## Analysis and Lessons

Our cross-domain evaluation reveals several key insights about hierarchical text classification methods. To better understand these findings, we conduct in-depth analysis and extensive additional experiments investigating: the surprising effectiveness of methods outside their original domains (Sect. 5.1), the potential of combining submodules across domains (Sect. 5.2), and the impact of various design choices through controlled experiments, including domain-specific language models (Sect. 5.3), document length handling strategies (Sect. 5.4), training data size variations (Sect. 5.5), and label hierarchy initialization methods (Sect. 5.6). Through these systematic analyses and ablation studies, we aim to provide practical guidance for selecting and adapting methods across different domains.

### State-of-the-Art Performance Often Comes from Other Domains

Our cross-domain evaluation reveals interesting patterns in how methods perform beyond their original domains as shown in Table 7:

**Table 7:** Summary of SOTA methods and cross-domain top-performer changes

Dataset	Domain	SOTA claimed (status)	New top performers		
			1	2	3
WOS-141	Scientific	HILL$^{\mathsf{{SN}}}$ (Y)	HILL$^{\mathsf{{SN}}}$	PLM-ICD$^{\mathsf{{M}}}$	HiAGM-TP$^{\mathsf{{SN}}}$
NYT-166	News	HILL$^{\mathsf{{SN}}}$ (**N**)	XR-Trans$^{\mathsf{{L}}}$	MatchXML$^{\mathsf{{L}}}$	PLM-ICD$^{\mathsf{{M}}}$
EurLex-3985	Legal	MatchXML$^{\mathsf{{L}}}$ (Y)	XR-Trans$^{\mathsf{{L}}}$	MatchXML$^{\mathsf{{L}}}$	PLM-ICD$^{\mathsf{{M}}}$
EurLex-DC-410	Legal	–	MatchXML$^{\mathsf{{L}}}$	XR-Trans$^{\mathsf{{L}}}$	PLM-ICD$^{\mathsf{{M}}}$
SciHTC-83	Scientific	HR-SciBERT$^{\mathsf{{SN}}}$ (**N**)	MatchXML$^{\mathsf{{L}}}$	THMM$^{\mathsf{{P}}}$	XR-Trans$^{\mathsf{{L}}}$
SciHTC-800	Scientific	–	MatchXML$^{\mathsf{{L}}}$	XR-Trans$^{\mathsf{{L}}}$	THMM$^{\mathsf{{P}}}$
MIMIC3-3681	Medical	PLM-ICD$^{\mathsf{{M}}}$ (Y)	PLM-ICD$^{\mathsf{{M}}}$	XR-Trans$^{\mathsf{{L}}}$	MatchXML$^{\mathsf{{L}}}$
USPTO2M-632	Patent	THMM$^{\mathsf{{P}}}$ (**N**)	MatchXML$^{\mathsf{{L}}}$	THMM$^{\mathsf{{P}}}$	FlatBERT$^{\mathsf{{P}}}$

Y: SOTA maintained; **N: SOTA lost**; $^{\mathsf{{SN}}}$ Methods originally evaluated on Scientific or News domain; $^{\mathsf{{L}}}$ Methods originally evaluated on Legal domain; $^{\mathsf{{M}}}$ Medical domain methods; $^{\mathsf{{P}}}$ Patent domain methods

**WOS-141**: HILL maintains its leading position, but the top three performers now include PLM-ICD, originally designed for medical coding. **NYT-166**: Methods from the legal domain, XR-Transformer and MatchXML, have surpassed the previous leader, HILL. PLM-ICD from the medical domain also ranks among the top performers. EurLex-*: MatchXML and XR-Transformer continue to excel in their native legal domain. Notably, PLM-ICD, from the medical domain, consistently ranks third, indicating that document handling techniques from medical texts can enhance legal document classification. SciHTC-*: The original leader, HR-SciBERT-mt, has been overtaken by methods from other domains, including MatchXML and XR-Transformer from the legal domain, THMM from the patent domain. **MIMIC3-3681**: PLM-ICD retains its top status in the medical domain, while legal domain methods, XR-Transformer and MatchXML, achieve competitive performance. **USPTO2M-632**: MatchXML, a method from the legal domain, surpasses the previous leader, THMM.

*Hybrid dominance in rankings* The top-three performers for each dataset all span multiple domains, suggesting that effective hierarchical text classification strategies are often domain-agnostic. Methods originally designed for extreme multi-label classification (MatchXML, XR-Transformer) show particularly strong cross-domain generalization, while medical domain innovations in document handling (PLM-ICD) prove valuable across various technical domains.

**Fig. 3:**
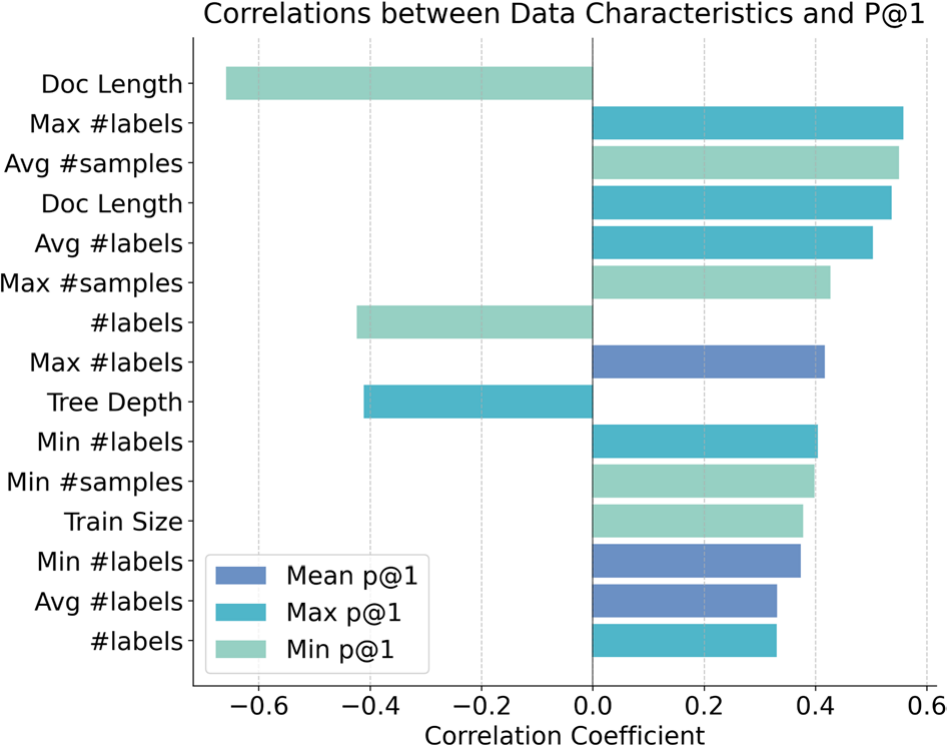
Correlations between dataset characteristics and model performance (precision@1). Only correlations with absolute values greater than 0.3 are shown, with features sorted top-down by absolute correlation values. The y-axis shows dataset features, where “#labels” refer to the total number of distinct classes, “Max/Min/Avg #labels” refers to statistics about how many labels each document has, i.e., mean, max, min number of labels per document and similarly “Max/Min/Avg #samples” refers to statistics about how many training examples each label has. Different colored bars show three performance metrics: mean, maximum, and minimum precision@1 scores across all models

*Dataset characteristics matter more than domain specificity* To understand what truly drives model performance, we analyzed correlations between dataset features (e.g., document length, labels per sample, training size) and model performance metrics (mean, max, and min precision@1 across models). Figure 3 shows correlations with absolute values above 0.3.

The results show interesting patterns. *Document length* shows a strong divergent effect ($r = +0.535$ for max precision@1, $r = -0.657$ for min precision@1). Advanced models excel with longer documents, while baseline models perform poorly. Similarly, *label size* shows a divergent effect ($r = +0.329$ for max precision@1, $r = -0.423$ for min precision@1). *Per-sample label cardinality* demonstrates consistent positive impact for above-average models. The average, maximum, and minimum number of labels per sample positively correlate with maximum precision@1 and mean precision@1. *Label density* significantly benefits weak models. The average, maximum and minimum number of samples per label positively correlate with minimum precision@1 ($r=0.549$ between avg samples per label and min precision@1). *Hierarchical depth* negatively impacts top performers ($r = -0.410$ for max precision@1).

These findings challenge the common practice of developing and evaluating methods within single domains. It also highlights the need for thorough cross-domain testing and unified evaluation to improve hierarchical text classification.

### Cross-domain Submodule Synthesis Enables Performance Gains

**Fig. 4:**
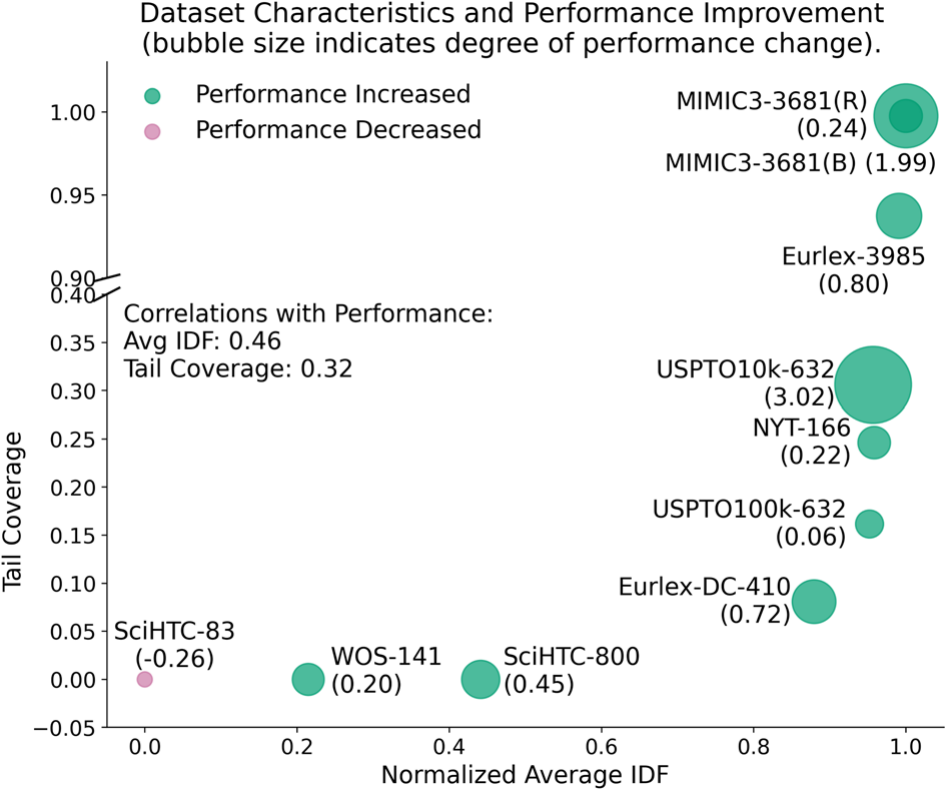
Performance changes from PLM-ICD to PLM-ICD+Label2Vec plotted against dataset characteristics. It shows that augmenting PLM-ICD with label semantic information is beneficial for datasets containing *diverse and rare label combinations*. The x-axis shows the average pattern IDF (measuring label combination diversity, see definition 1), and the y-axis shows tail pattern coverage (proportion of samples with rare label combinations, see definition 2). Each point represents a dataset, with larger improvements (shown by point size) occurring in datasets with both high IDF and tail coverage. MIMIC3-3681 results are shown for both BERT (B) and RoBERTa-pm (R) encoders, where RoBERTa-pm is the original text encoder used by PLM-ICD

Beyond revealing cross-domain performance patterns, our framework enables a critical capability: systematically identifying and combining complementary architectural strengths across domains to create improved models. This validates the framework’s practical utility as outlined in our methodology (Sect. 4.1).

Our benchmark (Sect. 4) revealed that PLM-ICD (Medical domain) excels at handling long documents but lacks explicit label semantics, while MatchXML (Legal domain) captures label semantics through its Label2Vec submodule. Guided by our framework, we synthesized a hybrid model by integrating Label2Vec into PLM-ICD. Implementation details are in “Appendix 9”.

Table 8 shows that this combination improves performance across most datasets, achieving new state-of-the-art results on MIMIC3-3681. We investigate the results by analyzing datasets using the following two metrics based on *label patterns*, i.e. unique sets of labels assigned to individual samples.

1. Average pattern IDF: $\text {IDF}(p) = \frac{1}{|P|}\sum _{p \in P}\log \frac{N}{f_p + 1}$ where *N* is number of total samples, $f_p$ is frequency of pattern *p*, and *P* is the set of unique label patterns. Higher values indicate more diverse label combinations.
2. Tail pattern coverage: $\frac{\sum _{p: f_p \le \theta } f_p}{\sum _{p \in P} f_p}$ where $\theta =3$, measuring the proportion of samples with rare label combinations.

The improvements positively correlate with both measurements. Figure 4 visualizes such correlations. Larger performance gains occur in datasets with both high IDF and tail coverage, suggesting our synthesized method is particularly effective for datasets with many rare label combinations.

This finding validates the core premise of our paper: the unified framework enables systematic identification of complementary strengths across domains, leading to improved models. Importantly, this capability emerges naturally from our cross-domain evaluation. Without evaluating PLM-ICD on diverse datasets, we would not have discovered its limitation (lack of label semantics), and without evaluating MatchXML cross-domain, we would not have identified its transferable strength (Label2Vec). This demonstrates that cross-domain evaluation is not merely diagnostic but generative: it reveals novel architectural combinations that would remain hidden in domain-isolated research.

**Table 8:** Precision@1 comparison of PLM-ICD and PLM-ICD+L2V

Method	WOS -141	NYT -166	EurLex -3985	EurLex-DC -410	SciHTC -83	SciHTC -800	MIMIC3 -3681 BERT	MIMIC3 -3681 RoBERTa-pm	USPTO 10k -632	USPTO 100k -632
PLM-ICD	89.88	96.03	94.77	87.36	61.40	38.40	87.13	90.77	60.74	73.21
+L2V	90.08	96.25	95.57	88.08	61.14	38.85	89.12	**91**.01*	63.76	73.27

*New state-of-the-art result on MIMIC3-3681

### Domain-Specific LLMs are Beneficial, Especially for Simpler Models and Low-Resource Settings

**Fig. 5:**
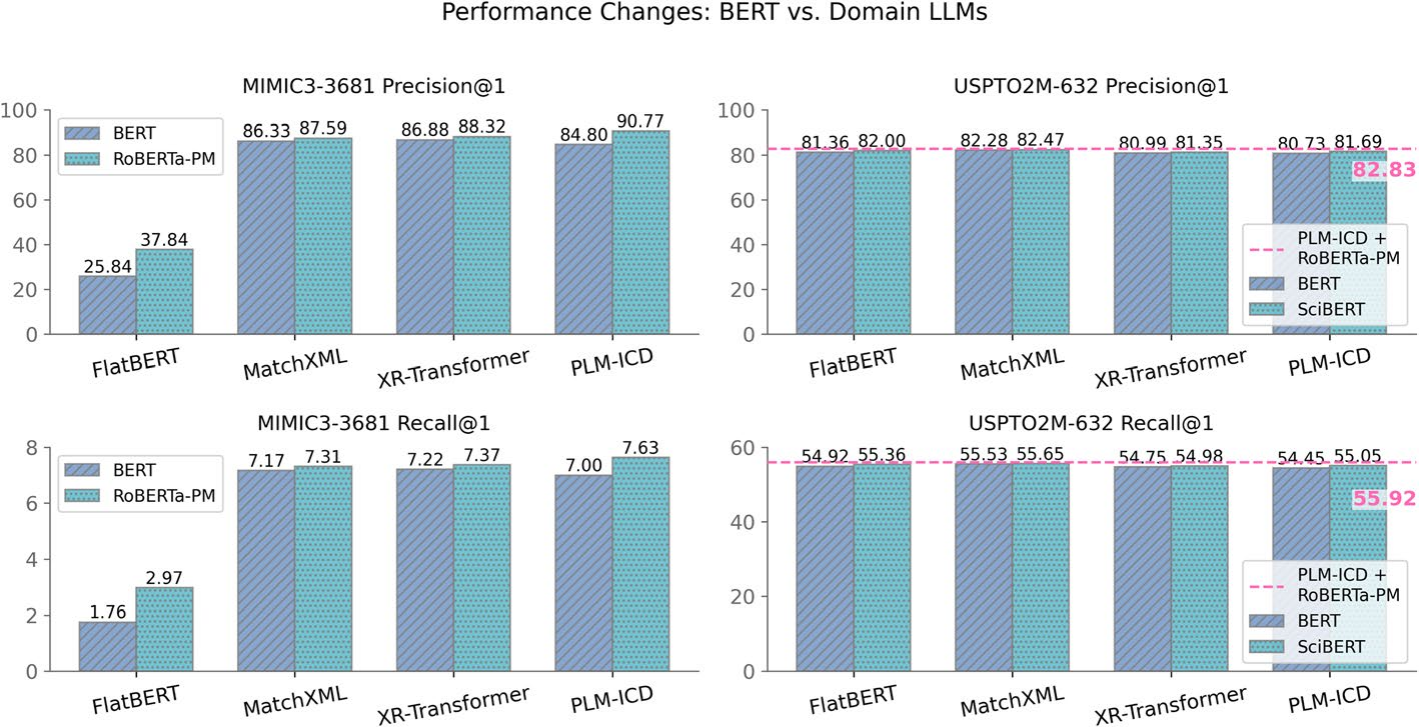
P/R@1 changes from BERT to domain-specific LLMs on MIMIC3-3681 and USPTO2M-632. The paired bars show performance improvements when switching from BERT to domain-specific LLMs. Larger gains are seen on MIMIC3-3681 compared to USPTO2M-632, especially for simpler architectures like FlatBERT. The horizontal dashed line indicates a *new state-of-the-art* achieved by PLM-ICD using RoBERTa-PM (a medical LLM) on USPTO2M-632 (patent), surprisingly outperforming SciBERT (a scientific LLM)

Among methods using language models as text encoders, most use bert-base-uncased (Devlin, 2018) by default, while PLM-ICD and THMM employ domain-specific models (RoBERTa-PM (Lewis et al., 2020) and SciBERT (Beltagy et al., 2019)) on their respective domains, following the original papers. Given PLM-ICD’s superior performance on MIMIC3-3681, we investigate whether this advantage comes from its architecture or its domain-specific encoder, as well as the impact of domain-specific LLMs on other methods, with results shown in Fig. 5.

All methods using domain-specific LLMs outperform the default bert-base-uncased encoder, and the performance gap is more significant for FlatBERT and MIMIC3-3681. Two conclusions can be drawn: (1) simpler architectures like FlatBERT benefit more from careful language model selection than complex ones, and (2) the impact of language model choice may diminish with larger training datasets, as evidenced by the smaller performance changes on USPTO2M-632.

PLM-ICD’s performance drops significantly when switching from RoBERTa-PM to BERT on MIMIC3-3681, losing its status as state-of-the-art. This indicates that the domain-specific encoder plays a crucial role in its superior performance, while using SciBERT on USPTO2M-632 shows slight improvements.

*A surprising new state of the art with a ‘mismatched’ cross-domain LLM* In a surprising cross-domain application, using RoBERTa-PM (a medical domain LLM) for PLM-ICD on USPTO2M-632 (a patent dataset) improves performance (80.73/54.45 to 82.83/55.92) even more than using SciBERT (a scientific domain LLM), surpassing the original SOTA from MatchXML. This counterintuitive success of using a medical LLM for patent classification suggests the improvement may stem from RoBERTa-PM’s larger vocabulary rather than domain-specific knowledge. This finding hints at potential benefits of cross-domain LLM applications. Nonetheless, the confirmation of this hypothesis is left as future work.

### Long Document Handling is Crucial for Medical Text

Text encoders generally face challenges with long documents, whether due to memory constraints in RNNs or token length limitations in pretrained language models (e.g., 512 tokens for BERT-family models). While most methods simply truncate documents to handle these limitations, our experiments on MIMIC3-3681 reveal the significant impact of long document handling strategies on model performance, shown in Table 9.

**Table 9:** Impact of document handling strategies on MIMIC3-3681

Method	Change	Performance (P@1/R@1) change
PLM-ICD	Full doc $\rightarrow$ truncated	90.77/7.63 $\Downarrow$ $-$ **4.48**/$-$ **0.46** 86.29/7.17
XR-Transformer	Truncated $\rightarrow$ mean pool	88.32/7.37 $\Downarrow$ $-$ **1.94**/$-$ **0.19** 86.38/7.18

PLM-ICD’s full document processing outperforms truncation, while naive mean pooling over 512-token segments in XR-Transformer underperforms simple truncation, suggesting effective long document handling requires sophisticated design

PLM-ICD’s sophisticated handling of full documents proves beneficial, while our attempt to enhance XR-Transformer beyond its default truncation by averaging embeddings across 512-token segments actually hurts performance. This counter-intuitive result suggests that averaging across document segments may dilute important signals, and effective long document strategies need careful design, potentially incorporating domain knowledge and label-aware mechanisms.

### Simple Models are Good Enough Sometimes, But More Easily Affected by the Data Size

Our cross-domain evaluation shows that FlatBERT performs poorly on most datasets but achieves competitive results on USPTO2M-632. We hypothesize this exception is due to USPTO2M’s large training set (1.9M samples), which compensates for FlatBERT’s simple architecture.

To investigate the impact of training data *quantity* on model performance, particularly for simpler versus more sophisticated architectures, we created reduced-size versions of the USPTO2M-632 training data. Specifically, we generated two smaller training sets: **USPTO100k-632** (containing 100,000 samples) and **USPTO10k-632** (containing 10,000 samples).

Crucially, these smaller training sets were created by performing stratified sampling from the original 1.9M training samples of USPTO2M-632. We employed stratified sampling *not* to alter the inherent class imbalance of the dataset or to try and match the test set distribution, but specifically to ensure that the relative proportions of classes within our newly created smaller training sets (100k and 10k samples) remained representative of the class proportions found in the original, full 1.9M training set. By keeping the class proportions consistent (though scaled down), we can more confidently attribute performance changes to the sheer volume of training data rather than to shifts in class imbalance. The original test set for USPTO2M-632 was, of course, kept entirely unchanged across all these experiments to provide a stable and consistent benchmark for evaluation. The results of training various models on these differently sized training sets are presented in Table 10.

**Table 10:** Precision/Recall@1 for varied training sizes of USPTO*-632

Method	2M	100k	10k
MatchXML	82.28/55.53	76.23/51.38 **(** $-$ **6.05/** $-$ **4.15)**	65.05/43.71 **(** $-$ **11.18/** $-$ **7.67)**
XR-Transformer	80.99/54.75	74.94/50.60 **(** $-$ **6.05/** $-$ **4.15)**	66.34/44.57 **(** $-$ **8.60/** $-$ **6.03)**
HILL	ET	74.59/50.25	65.30/43.95 **(** $-$ **9.29/** $-$ **6.30)**
HiAGM-TP	ET	71.19/47.90	58.06/39.04 **(** $-$ **13.13/** $-$ **8.86)**
PLM-ICD	80.73/54.45	73.21/49.32 **(** $-$ **7.52/** $-$ **5.13)**	60.74/40.76 **(** $-$ **12.47/** $-$ **8.56)**
FlatBERT	82.00/55.36	69.34/46.54 **(** $-$ **12.66/** $-$ **8.82)**	17.93/11.78 **(** $-$ **51.41/** $-$ **34.76)**
THMM	81.83/55.26	72.00/48.37 **(** $-$ **9.83/** $-$ **6.89)**	36.98/23.81 **(** $-$ **35.02/** $-$ **24.56)**

Simple models like FlatBERT suffer catastrophic drops with reduced data, while sophisticated architectures like XR-Transformer maintain reasonable performance

The results confirm our hypothesis: FlatBERT’s performance drops dramatically with reduced training data, from competitive (82.00/55.36) to catastrophic (17.93/11.78). In contrast, methods with more sophisticated architectures like XR-Transformer maintain reasonable performance even with only 10k samples. This explains FlatBERT’s inconsistent performance across datasets: it requires large-scale training data to compensate for its simple architecture.

### Prior Knowledge of Label Hierarchy for Probabilistic Label Tree is Somewhat Beneficial

We investigate the impact of different initialization methods of the Probabilistic Label Tree (PLT) in XR-Transformer, which controls how the output space is initially segmented. We compare three approaches: PIFA (the original method using hierarchical clustering based on training samples), Gold (using the ground-truth taxonomy tree), and random initialization. Our experiments show minimal differences between PIFA and Gold, though random initialization leads to decreased performance (see precision and recall details in Tables 19 and 20). These results suggest that while prior knowledge of label hierarchies may provide some benefit, the advantage may be modest.

## Conclusion

This paper proposes a unified framework for hierarchical multi-label text classification and conducts a comprehensive cross-domain analysis of state-of-the-art methods. Our evaluation reveals several important **insights**. *First*, top performance often comes from methods developed for other domains, which challenges the common practice of domain-specific method development. For instance, we achieved new state-of-the-art results on NYT-166, SciHTC-83, and USPTO2M-632 using methods originally proposed for other domains. *Second*, the effectiveness of methods depends more on dataset characteristics, such as label patterns, document length, and training size, than on the domain itself. *Third*, combining architectural innovations from different domains can create more robust models and even achieve new state-of-the-art results, as demonstrated by our experiments in Sect. 5.2.

Other key **findings** include: domain-specific language models significantly improve performance, especially for simpler architectures and challenging datasets; sophisticated document handling is crucial for long texts like medical records; simple architectures can achieve competitive performance but require substantial training data; and prior knowledge of label hierarchies provides modest benefits.

Our study has several **limitations** that highlight opportunities for future research. On the *resource* side, computational constraints limit our evaluation to a subset of available methods and datasets, potentially missing valuable insights from other approaches. This also affects our hyperparameter optimization, possibly underestimating some methods’ full potential. Our unified evaluation framework, while enabling fair comparisons across methods, may yield results that differ from those reported in original papers due to standardized preprocessing and evaluation settings.

Regarding *methodological scope*, our focus on end-to-end architectures excludes standalone components like data augmentation or post-processing methods that could provide complementary benefits. For example, as discussed in Sect. 4.2.3, our baseline approach to data splitting did not employ specialized techniques to guarantee representation of extremely rare classes, nor did we implement label filtering or advanced upsampling techniques for such classes beyond what is inherent in the surveyed methods themselves. A more systematic exploration of such pre-processing and data augmentation strategies would be valuable further work. Additionally, methods for semi-supervised, unsupervised, or zero-shot approaches remain to be explored. Furthermore, we focus primarily on neural approaches to hierarchical text classification, without discussing symbolic or hybrid symbolic-neural approaches that might offer different trade-offs in terms of performance and interpretability. Future work should systematically evaluate these dimensions alongside performance metrics to provide a more comprehensive assessment of hierarchical text classification methods.

Regarding the *datasets*, our empirical evaluation concentrated on datasets with moderately large to large label spaces (all containing over 80 labels). This reflects the typical application scenarios for hierarchical text classification methods, where the complexity and potential benefit of leveraging the hierarchy are most pronounced. The performance and applicability of these methods on tasks with significantly smaller, potentially simpler hierarchies or alternative strategies for managing extreme label sparsity on the current datasets remain outside the scope of this specific investigation. Furthermore, our primary evaluation relied on standard or random splits due to common practice and scalability concerns with stratification in the field.

Important considerations such as model *interpretability*, *explainability*, and *energy consumption* were not addressed in this study. Interpretability and explainability are particularly crucial in domains like healthcare and legal applications, where understanding model decisions is an essential requirement. The computational efficiency and carbon footprint of these methods, especially those using large language models, represent important practical considerations for real-world deployment.

Several promising research **directions** emerge from our findings. *First*, the success of combining PLM-ICD with Label2Vec suggests potential in systematically exploring other cross-domain architectural combinations. *Second*, the significant impact of document handling strategies on medical texts indicates a need for more sophisticated approaches that can effectively process long documents while maintaining computational efficiency. *Third*, developing methods that can maintain performance with limited training data or computational resources would increase practical applicability across domains. This includes further research into effective data augmentation, oversampling, and other techniques for mitigating the impact of long-tail distributions and data sparsity for rare labels, potentially integrated within the submodules of our proposed framework. *Fourth*, recent advances in large language models suggest opportunities for effective zero-shot and few-shot classification with minimal domain-specific training data. *Lastly*, extending evaluation to emerging domains like skill tagging (Li et al., 2023b, a) could reveal additional insights about method generalization and domain-specific challenges.

Looking ahead, the key takeaway of our study is that it is valuable for researchers and practitioners to consider possibilities beyond domain boundaries in hierarchical text classification. Rather than defaulting to domain-specific solutions, we should explore methods that have succeeded elsewhere. This shift in approach could accelerate progress and lead to more robust and effective classification systems across all domains.

## Data Availability

Data is provided within the manuscript or supplementary information files.

## Appendix A: Details of the Submodules Used in Recent Methods

Table 11 shows the submodules used in recent methods.

**Table 11:** Submodules used in recent methods

Method	Text encoder	Label encoder	Out space seg	Text-label info fusion	Training objectives	Score prediction	Prediction refinement
XR-Transformer	LLM$^{0}$ &TF-IDF	PIFA	PLT	–	(Multi-stage) CLS loss$^{1}$+ Contrastive (MAN+TFN)	MS Linear	MS ranker
MatchXML	LLM$^{0}$ &TF-IDF	Label2Vec	PLT	Embedding alignment	(Multi-stage) CLS loss + Contrastive (MAN+TFN) + Alignment loss	MS Linear	MS ranker
HILL	LLM$^{0}$	Coding Tree	-	Structure encoder$^{2}$	CLS loss + NT-Xent contrastive loss	Linear	–
HiAGM-TP	GRU + CNN	Tree-LSTM/GCN	–	Structure encoder$^{3}$	CLS loss with regularization	Linear	–
HiAGM-LA	GRU + CNN	Tree-LSTM/GCN	–	Structured label attn	CLS loss with regularization	Linear	–
PLM-ICD	LLM$^{0}$	–	–	Segmented text with label-aware attn	CLS loss	Linear	–
FlatBERT$^{4}$	LLM$^{0}$	–	–	–	CLS loss	Linear	–
THMM	LLM$^{0}$	–	Original taxonomy	Parent-kid representation concatenation	CLS loss	An MLP per class	–
HR-SciBERT-mt	LLM$^{0}$	–	Original taxonomy	Parent-kid parameter sharing	CLS loss + Keyword prediction loss	An MLP per class	–
X-Transformer	LLM & TF-IDF	PIFA	PLT	–	CLS loss + Contrastive (MAN+TFN)	Linear	1-stage ranker
XR-Linear	TF-IDF	PIFA	PLT	–	MS CLS loss + Contrastive (MAN+TFN)	MS Linear	MS ranker
AttentionXML	LSTM	PIFA	PLT	label attn	(Multi-Stage) CLS	MS Linear	–
Bonsai	TF-IDF	PIFA	PLT	–	(Multi-Stage) CLS	MS Linear	–
HVHMC	GCN (word-doc)	GCN(label-word) inter+intra level dependency	–	repr concat	local + global CLS	level-wise + all cat	–
HGCLR	LLM	Graphnomer	–	label guided positive sample gen	CLS + NT-Xent contrastive	Linear	–
HBGL	LLM	LLM with custom pretraining task	–	Repr concat + custom attn mask	Multi-stage CLS + label mask pretraining	level-wise	–
HARNN	LSTM	dense vec	–	HAM	local + global CLS	level-wise + all cat	–
HTrans	GRU + modified outputs	–	Original taxonomy	Parent-kid parameter sharing	CLS	An MLP per class	–
PARL	LLM (bi+cross) + GraphSAGE+ BM25	LLM	–	Bi-enc + Cross-enc	Ranknet loss	L2R	–
HyperIM	Hyperbolic	Hyperbolic	–	Label-aware doc repr (geodesic distance)	CLS loss + Contrastive	emb sim	–
LA-HCN	LSTM	dense vec	–	Level-wise label attn+ label-component assoc.	local + global CLS	combine local+global	–
HCSM	CNN+LSTM	w2v	–	HARNN+ HON-LSTM+ HBiCaps	Margin loss	1 cap for 1 class	–
HiLAP	CNN	dense vec	Original taxonomy	–	Neg discounted cum rewards	Policy network	–
ExMLDS	TF-IDF+ ADMM projection	skip-gram + SPPMI factorization	–	ADMM projection	CLS + skig-gram neg+ label co-occurance reg	emb similarity	–
GalaXC	Fixed pre-trained	GNN + label attention	ANNS	Joint doc-label graph	CLS loss + neg Contrastive	emb sim	–
EcLARE	TF-IDF	GCN + label attention	Graph-Assisted Multi-label Expansion (GAME)	–	CLS loss + neg Contrastive + spectral reg	emb sim	–
*Plug-in Methods (can be combined with methods above)*							
*Training objective enhancements*							
DepReg	Adds dependency regularization term to training objectives						
*Label space processing*							
PS-PLT	Enhances PLT with propensity scoring for better label space segmentation						
*Prediction refinement*							
Capsule Net	Replaces standard prediction with capsule network + label correlation restrictions						
CorNet	Adds correlation-based prediction refinement layer						
*Data augmentation*							
GANDALF	Graph-based data augmentation for long-tail labels						
REMIDIAL	Data augmentation through label correlation patterns						

$^{0}$LLM: Large Language Model, e.g. BERT, RoBERTa, etc. Specifically, we use BERT as the default LLM encoder except for the following cases: RoBERTa-PM for PLM-ICD on MIMIC3-3681 and SciBERT for THMM on USPTO2M-632 following the original papers

$^{1}$Classification loss for multi-label classification, depending on the specific loss function used, most commonly the binary cross-entropy loss

$^{2}$A custom defined structure encoder that propagates information from the leaf nodes to the root node of the coding tree

$^{3}$Tree-LSTM or GCN, corresponding to the label encoder.

$^{4}$PatentBERT in the original paper due to its application in patent classification. We rename it as FlatBERT for generalization

## Appendix B: Additional Experiment Results

Here we present precision and recall scores at varied *k* values as well as f1 scores.

### Impact of Different k Values

While the main text focuses on Precision/Recall@1, analyzing model behaviors at $k=3, 5, 8, 10$ reveals additional insights into multi-label prediction capabilities (Tables 12, 13, 14, 15, 16, 17, 5, 6).

*Precision-Recall Trade-offs* All datasets show precision decreases and recall increases as *k* grows, but with notably different magnitudes. WOS shows dramatic precision drops ($\sim$ 70 percentage points from $k=1$ to $k=10$, e.g., HILL: 90.81 $\rightarrow$ 19.31) due to its having only two labels per document. MIMIC3 exhibits more gradual degradation ($\sim$ 25 points, e.g., PLM-ICD: 90.77 $\rightarrow$ 67.11) for its high label cardinality. EurLex-3985 demonstrates the most stable precision metrics with only $\sim$ 6–7 point drops from $k=1$ to $k=5$ (XR-Transformer: 97.67 $\rightarrow$ 90.91).

*Model Ranking Changes* Analysis of model rankings across $k=1,3,5,8,10$ reveals several consistent patterns and notable shifts. Some models maintain stable leadership positions: XR-Transformer consistently ranks first on NYT-166 and EurLex-3985, HILL maintains first place on WOS-141, and PLM-ICD leads on MIMIC3-3681 across all *k* values. However, significant ranking changes emerge on certain datasets. PLM-ICD shows marked improvement on SciHTC-83, climbing from rank 4 ($k=1$) to rank 1 ($k=8,10$), while HILL demonstrates similar improvement on EurLexDC-410, advancing from rank 4 to rank 1. Conversely, some models experience performance degradation at higher *k* values: MatchXML drops from rank 1 to rank 5 on SciHTC-83, and XR-Transformer declines from rank 3 to rank 7 on the same dataset. The most stable rankings across *k* values are observed for XR-Transformer on NYT-166, EurLex-3985, and USPTO2M-632, HILL on WOS-141, and PLM-ICD on MIMIC3-3681.

**Table 12:** Cross-domain Precision@3 scores and ranks

Method	$^{4}$WOS -141	$^{4}$NYT -166	$^{4}$EurLex -3985	$^{4}$EurLexDC -410	$^{4}$SciHTC -83	$^{4}$SciHTC -800	$^{4}$MIMIC3 -3681	$^{4}$USPTO2M -632
MatchXML	57.28 - **5**	84.67 - **2**	94.36 - **2**	38.92 - **2**	39.90 - **1**	25.79 - **1**	79.42 - **3**	44.22 - **4**
XR-Transformer	57.65 - **4**	85.16 - **1**	94.67 - **1**	39.02 - **1**	39.35 - **5**	25.15 - **2**	80.04 - **2**	42.75 - **5**
HILL	60.23 - **1**	81.83 - **4**	92.27 - **3**	37.94 - **3**	36.56 - **8**	21.84 - **7**	71.31 - **4**	ET
HiAGM-TP	60.10 - **2**	73.76 - **7**	87.19 - **5**	37.19 - **5**	36.93 - **7**	23.35 - **6**	EM	ET
PLM-ICD	59.17 - **3**	84.04 - **3**	91.37 - **4**	37.81 - **4**	39.65 - **3**	24.04 - **4**	85.64 - **1**	44.57 - **3**
FlatBERT	34.81 - **7**	75.23 - **6**	42.53 - **7**	6.55 - **7**	38.99 - **6**	23.95 - **5**	19.46 - **5**	44.85 - **2**
THMM	57.24 - **6**	81.74 - **5**	54.32 - **6**	22.17 - **6**	39.83 - **2**	24.63 - **3**	EM	45.20 - **1**
HR-SciBERT-mt	ET	ET	ET	ET	39.44 - **4**	ET	ET	ET

**Table 13:** Cross-domain Recall@3 scores and ranks

Method	WOS -141	NYT -166	EurLex -3985	EurLexDC -410	SciHTC -83	SciHTC -800	MIMIC3 -3681	USPTO2M -632
MatchXML	85.93 - **5**	53.97 - **2**	23.36 - **2**	93.28 - **2**	66.70 - **1**	50.30 - **1**	19.22 - **3**	78.23 - **4**
XR-Transformer	86.47 - **4**	54.36 - **1**	23.46 - **1**	93.62 - **1**	65.83 - **4**	48.94 - **2**	19.38 - **2**	76.19 - **5**
HILL	90.34 - **1**	51.33 - **5**	22.79 - **3**	91.62 - **3**	60.98 - **8**	42.26 - **7**	17.01 - **4**	ET
HiAGM-TP	90.16 - **2**	47.09 - **6**	21.49 - **5**	89.89 - **5**	61.41 - **7**	45.05 - **6**	EM	ET
PLM-ICD	88.75 - **3**	53.45 - **3**	22.60 - **4**	90.96 - **4**	66.34 - **3**	46.44 - **5**	20.94 - **1**	78.70 - **3**
FlatBERT	52.22 - **7**	46.08 - **7**	9.87 - **7**	18.40 - **7**	65.41 - **6**	47.27 - **4**	4.15 - **5**	79.04 - **2**
THMM	85.86 - **6**	51.42 - **4**	12.82 - **6**	56.37 - **6**	66.54 - **2**	47.59 - **3**	EM	79.44 - **1**
HR-SciBERT-mt	ET	ET	ET	ET	65.78 - **5**	ET	ET	ET

**Table 14:** Cross-domain Precision@5 scores and ranks

Method	WOS -141	NYT -166	EurLex -3985	EurLexDC -410	SciHTC -83	SciHTC -800	MIMIC3 -3681	USPTO2M -632
MatchXML	36.29 - **5**	72.24 - **2**	90.69 - **2**	23.87 - **2**	27.73 - **4**	18.52 - **1**	72.77 - **3**	29.75 - **4**
XR-Transformer	36.67 - **4**	72.58 - **1**	90.91 - **1**	24.01 - **1**	27.30 - **6**	17.93 - **3**	73.32 - **2**	28.60 - **5**
HILL	37.53 - **1**	69.69 - **5**	88.94 - **3**	23.70 - **3**	25.75 - **8**	15.97 - **7**	64.29 - **4**	ET
HiAGM-TP	37.39 - **2**	61.81 - **7**	82.97 - **5**	23.22 - **5**	26.47 - **7**	17.11 - **6**	EM	ET
PLM-ICD	37.10 - **3**	71.89 - **3**	87.11 - **4**	23.40 - **4**	27.97 - **2**	17.80 - **4**	80.02 - **1**	30.46 - **3**
FlatBERT	23.77 - **7**	64.12 - **6**	37.70 - **7**	4.15 - **7**	27.56 - **5**	17.52 - **5**	14.38 - **5**	30.71 - **2**
THMM	35.73 - **6**	69.73 - **4**	47.55 - **6**	14.79 - **6**	28.05 - **1**	18.05 - **2**	EM	30.92 - **1**
HR-SciBERT-mt	ET	ET	ET	ET	27.95 - **3**	ET	ET	ET

**Table 15:** Cross-domain Recall@5 scores and ranks

Method	WOS -141	NYT -166	EurLex -3985	EurLexDC -410	SciHTC -83	SciHTC -800	MIMIC3 -3681	USPTO2M -632
MatchXML	90.72 - **5**	66.57 - **2**	37.20 - **2**	94.74 - **2**	76.71 - **4**	59.79 - **1**	28.58 - **3**	84.44 - **4**
XR-Transformer	91.67 - **4**	66.88 - **1**	37.31 - **1**	95.31 - **1**	75.53 - **6**	57.67 - **3**	28.80 - **2**	81.97 - **5**
HILL	93.83 - **1**	63.00 - **5**	36.39 - **3**	94.20 - **3**	71.33 - **8**	51.54 - **7**	24.94 - **4**	ET
HiAGM-TP	93.48 - **2**	57.34 - **6**	33.81 - **5**	92.76 - **5**	73.01 - **7**	55.22 - **6**	EM	ET
PLM-ICD	92.75 - **3**	66.04 - **3**	35.67 - **4**	93.08 - **4**	77.41 - **2**	57.43 - **4**	31.67 - **1**	85.93 - **3**
FlatBERT	59.41 - **7**	56.46 - **7**	14.53 - **7**	18.99 - **7**	76.40 - **5**	57.35 - **5**	5.19 - **5**	86.40 - **2**
THMM	89.33 - **6**	63.77 - **4**	18.61 - **6**	61.25 - **6**	77.58 - **1**	58.17 - **2**	EM	86.76 - **1**
HR-SciBERT-mt	ET	ET	ET	ET	77.21 - **3**	ET	ET	ET

**Table 16:** Cross-domain Precision@8 scores and ranks

Method	WOS -141	NYT -166	EurLex -3985	EurLexDC -410	SciHTC -83	SciHTC -800	MIMIC3 -3681	USPTO2M -632
MatchXML	23.36 - **5**	60.60 - **2**	83.40 - **2**	15.07 - **3**	19.03 - **5**	13.10 - **1**	63.90 - **3**	19.83 - **4**
XR-Transformer	23.56 - **4**	61.04 - **1**	83.55 - **1**	15.16 - **2**	18.67 - **6**	12.63 - **4**	64.22 - **2**	19.02 - **5**
HILL	23.96 - **1**	58.32 - **4**	82.18 - **3**	15.19 - **1**	17.91 - **8**	11.56 - **7**	55.18 - **4**	ET
HiAGM-TP	23.82 - **2**	50.89 - **7**	75.94 - **5**	14.84 - **5**	18.64 - **7**	12.43 - **6**	EM	ET
PLM-ICD	23.74 - **3**	60.53 - **3**	79.29 - **4**	14.90 - **4**	19.52 - **1**	12.95 - **3**	72.07 - **1**	20.60 - **3**
FlatBERT	16.84 - **7**	54.00 - **6**	31.73 - **7**	2.63 - **7**	19.19 - **4**	12.61 - **5**	8.99 - **5**	20.79 - **2**
THMM	23.02 - **6**	58.14 - **5**	39.24 - **6**	10.21 - **6**	19.43 - **3**	13.04 - **2**	EM	20.90 - **1**
HR-SciBERT-mt	ET	ET	ET	ET	19.50 - **2**	ET	ET	ET

**Table 17:** Cross-domain Recall@8 scores and ranks

Method	WOS -141	NYT -166	EurLex -3985	EurLexDC -410	SciHTC -83	SciHTC -800	MIMIC3 -3681	USPTO2M -632
MatchXML	93.42 - **5**	78.53 - **2**	54.08 - **2**	95.50 - **3**	83.87 - **5**	67.32 - **1**	38.82 - **3**	88.08 - **4**
XR-Transformer	94.24 - **4**	79.02 - **1**	54.18 - **1**	96.09 - **2**	82.29 - **6**	64.73 - **5**	39.01 - **2**	85.43 - **5**
HILL	95.83 - **1**	74.47 - **5**	53.21 - **3**	96.13 - **1**	79.13 - **8**	59.69 - **7**	33.24 - **4**	ET
HiAGM-TP	95.27 - **2**	67.05 - **7**	48.92 - **5**	94.46 - **5**	82.03 - **7**	64.26 - **6**	EM	ET
PLM-ICD	94.97 - **3**	78.18 - **3**	51.30 - **4**	94.47 - **4**	86.11 - **1**	66.85 - **3**	44.03 - **1**	90.54 - **3**
FlatBERT	67.38 - **7**	68.51 - **6**	19.65 - **7**	19.14 - **7**	84.71 - **4**	65.83 - **4**	5.19 - **5**	91.03 - **2**
THMM	92.09 - **6**	75.55 - **4**	24.37 - **6**	66.87 - **6**	85.68 - **3**	67.07 - **2**	EM	91.33 - **1**
HR-SciBERT-mt	ET	ET	ET	ET	85.92 - **2**	ET	ET	ET

### F1 Scores (Micro/Macro)

Table 18 reports comparisons of precision/recall@ different *k* values and micro/macro f1 scores. They are calculated using models’ top-10 predictions against the ground truth.

**Table 18:** Cross-domain F1 scores (micro/macro)

Method	WOS -141	NYT -166	EurLex -3985	EurLex-DC -410	SciHTC -83	SciHTC -800	MIMIC3 -3681	USPTO2M -632
MatchXML	31.46/25.04	62.37/41.41	68.09/27.55	21.45/23.17	26.63/17.68	18.96/10.50	46.25/12.57	27.18/20.50
XR-Transformer	31.69/25.17	62.82/43.10	68.23/30.11	21.58/20.06	26.10/17.27	18.25/9.63	46.37/13.47	26.07/19.45
HILL	32.18/31.60	59.85/27.73	67.42/34.98	21.73/22.44	25.19/16.68	16.99/9.67	39.32/11.93	ET
HiAGM-TP	32.00/23.75	52.30/28.69	61.98/25.68	21.25/21.40	26.39/17.83	18.25/8.11	EM	ET
PLM-ICD	31.82/36.43	62.16/45.57	61.62/14.03	21.20/29.46	27.37/19.86	18.74/7.72	52.82/20.94	28.41/22.36
FlatBERT	24.23/10.17	55.08/14.23	25.05/0.16	6.96/0.21	26.98/18.70	18.48/5.88	8.74/0.19	28.64/22.94
THMM	31.04/23.71	59.48/34.44	31.04/0.38	15.12/4.58	27.29/19.08	19.10/7.48	EM	28.77/23.89
HR-SciBERT-mt	ET	ET	ET	ET	27.30/20.78	ET	ET	ET

Values are presented as Micro-F1/Macro-F1. ET the method exceeded the time limit. EM the method exceeded the memory limit

### Dataset- and Method-Specific Patterns

*Hierarchy depth* strongly influences the performance of certain methods. Shallow hierarchies (like in WOS-141) are better handled by HILL, HiAGM-TP, and THMM than datasets with deeper hierarchies (EurLex-*, SciHTC-*). *Document length* and label structure are key factors because datasets with long documents and flat label structures (MIMIC3-3681, USPTO2M-632) seem to require specialized text processing approaches, which PLM-ICD handles effectively. Datasets with *label sparsity*, i.e. with low average samples per label (EurLex-*), benefit from robust label-aware mechanisms, where MatchXML, XR-Transformer, and PLM-ICD excel. *Sample abundance* can compensate for other complexities. For example, datasets with high sample counts per label (like SciHTC-83) enable even simpler methods to perform well, as demonstrated by THMM’s unexpected success.

On the method side, the *baseline* FlatBERT lags behind other methods but shows competitive results with sufficient training data (USPTO2M-632). *Transformer-based* methods (MatchXML, XR-Transformer) excel with large label spaces but show limitations in exploiting label redundancy. *Per node classification* methods face scalability issues, as HR-SciBERT-mt usually exceeds time limits.

### XR-Transformer Tree Initialization Variations

The results (Tables 19, 20) demonstrate that tree initialization methods have a small but consistent impact on XR-Transformer’s performance. Random initialization consistently performs slightly worse than both PIFA and Gold methods across most datasets, with performance gaps of 0.1–0.5 percentage points in both precision and recall. Gold initialization generally achieves the best results (e.g., 97.67% precision on EurLex-3985), followed closely by PIFA, while Random initialization ranks last. This suggests that leveraging either Gold or PIFA tree structures is beneficial, though the performance differences are modest.

**Table 19:** XR-Transformer tree initialization variations precision@1

Init tree	WOS-141	NYT-166	EurLex-3956	EurLex-3985	EurLexDC	SciHTC-83	SciHTC-800	MIMIC3-3681 (512)	USPTO2M-632
PIFA	86.49	96.64	87.83	97.51	89.80	61.53	40.69	88.32	80.99
Gold	86.39	96.70	88.06	97.67	89.86	61.58	40.76	88.03	81.19
Random	86.40	96.71	86.77	97.48	89.79	61.37	40.68	87.69	80.82

**Table 20:** XR-Transformer tree initialization variations recall@1

Init tree	WOS-141	NYT-166	EurLex-3956	EurLex-3985	EurLexDC	SciHTC-83	SciHTC-800	MIMIC3-3681 (512)	USPTO2M-632
PIFA	43.25	22.93	17.82	8.09	78.91	36.29	27.43	7.37	54.75
Gold	43.20	22.94	17.88	8.10	78.96	36.32	27.49	7.35	54.90
Random	43.20	22.95	17.62	8.08	78.91	36.21	27.42	7.31	54.66

### Exploratory Experiment with Stratified Splitting

To investigate the potential impact of stratified data splitting, we conducted an additional experiment on the EurLex-3956 dataset (commonly known as EurLex-4k). We compared the performance of the XR-Transformer model using two different train/test splitting strategies (80% train/validation, 20% test):

1. **Random Split** Our standard random partitioning used for EurLex datasets where no official full-text split was available.
2. **Stratified Split** A split generated using Iterative Stratification (Sechidis et al., 2011) applied to the label sets.

The results for Precision@k and Recall@k are presented in Table 21.

**Table 21:** Comparison of XR-Transformer performance on EurLex-3956 with random versus stratified train/test splits

Split type	P@1	R@1	P@3	R@3	P@5	R@5	P@10	R@10
Random split	87.83	17.82	74.84	44.59	61.64	60.00	38.22	73.17
Stratified split	89.06	18.10	76.41	45.59	63.71	62.08	40.06	76.71

The results indicate a marginal improvement in measured performance when using the stratified split compared to the random split for this specific dataset and model. For instance, P@1 increased by 1.23 points and R@1 by 0.28 points. While these improvements observed in this post-hoc exploratory experiment are positive, they are relatively small. Considering these findings alongside the significant computational effort and methodological complexities (detailed in Sect. 4.2.3) associated with universally applying and tuning stratification across all datasets and methods in our completed large-scale survey, our original splitting strategy was retained for the main paper to ensure broad comparability and reproducibility based on common practices.

## Appendix C: Implementation Details of the Validation Case Study

To validate our framework, we synthesized a hybrid model by integrating the Label2Vec submodule from MatchXML (Legal domain) into the PLM-ICD architecture (Medical domain).

*Architecture Integration* PLM-ICD originally uses a label-aware attention mechanism where label representations are learned as free parameters. In our hybrid model, we replace these free parameters with a semantic label encoder. Specifically, we use the Label2Vec submodule, which initializes label embeddings using word vectors (Word2Vec or FastText) derived from the label descriptions. These embeddings are then fine-tuned during end-to-end training.

*Training Configuration* We maintained the original training configuration of PLM-ICD to ensure a fair comparison. The model was trained using binary cross-entropy loss. For the text encoder, we utilized bert-base-uncased for general domain datasets and RoBERTa-pm for the MIMIC-III dataset. The Label2Vec module was initialized with pre-trained FastText embeddings.

## Footnotes

1 These state-of-the-art results are based on our unified evaluation framework. While we carefully reproduced previous methods following their original implementations, some results may differ from those reported in original papers due to differences in preprocessing, evaluation settings, or implementation details.

2 For example, given a hierarchy where A.1.1 is a leaf node under A.1, which is under A, a document could be labeled as: (1) A.1.1 in a leaf-only scheme, (2) A, A.1, A.1.1 in a full-path scheme, or (3) A, A.1.1 in a flexible scheme. For simplicity, we do not consider the impact of these labeling schemes on data preprocessing or model design.

3 The 2019 cutoff was chosen to achieve a balance between focusing on the most recent methods and including the most relevant and frequently cited methods.

4 HiAGM has two variants that differ in text-label fusion, and we select HiAGM-TP for benchmark evaluation. We rename PatentBERT to FlatBERT to reflect its architecture that processes labels without hierarchical information.

5 https://eur-lex.europa.eu/homepage.html.

6 https://op.europa.eu/en/web/eu-vocabularies.

7 The similarity metrics include simple Levenshtein distance ratio, best partial string matching, Levenshtein distance ratio after sorting words and that ratio between unique sorted word intersections.

8 https://tudatalib.ulb.tu-darmstadt.de/handle/tudatalib/2937.

9 https://github.com/yourh/AttentionXML/tree/master#datasets.

10 https://github.com/kk7nc/HDLTex.

11 The repository of NYT-166 https://catalog.ldc.upenn.edu/LDC2008T19 is not maintained at the time of our experiment, we contacted the authors who have conducted the experiments on this dataset to obtain the data.

12 https://github.com/JasonHoou/USPTO-2M.

13 Originally developed for extreme multi-label classification tasks, MatchXML and XR-Transformer are evaluated on textual datasets with a large number of labels, including legal domain, Wikipedia, and e-commerce.

14 We made exceptions for two cases that were completed before establishing these limits: HR-SciBERT-mt on SciHTC-83 and HiAGM-TP on EurLex-3985, both of which required over a week of training time. Their results are included for completeness.
